# Recent advances in metamaterials for simultaneous wireless information and power transmission

**DOI:** 10.1515/nanoph-2021-0657

**Published:** 2022-01-11

**Authors:** Shuncheng Tian, Xuanming Zhang, Xin Wang, Jiaqi Han, Long Li

**Affiliations:** Xidian University, Xi’an 710071, China; Xi’an University of Posts & Telecommunications, Xi’an 710121, China

**Keywords:** digital coding metasurface, metamaterials, metasurfaces, simultaneous wireless information and power transmission, wireless energy harvesting, wireless power transmission

## Abstract

In the last two decades, metamaterials and metasurfaces have introduced many new electromagnetic (EM) theory concepts and inspired contemporary design methodologies for EM devices and systems. This review focuses on the recent advances in metamaterials (MMs) for simultaneous wireless information and power transmission (SWIPT) technology. In the increasingly complex EM world, digital coding and programmable metamaterials and metasurfaces have enabled commercial opportunities with a broad impact on wireless communications and wireless power transfer. In this review, we first introduce the potential technologies for SWIPT. Then, it is followed by a comprehensive survey of various research efforts on metamaterial-based wireless information transmission (WIT), wireless power transmission (WPT), wireless energy harvesting (WEH) and SWIPT technologies. Finally, it is concluded with perspectives on the rapidly growing SWIPT requirement for 6G. This review is expected to provide researchers with insights into the trend and applications of metamaterial-based SWIPT technologies to stimulate future research in this emerging domain.

## 1 Introduction

**Figure 1:**
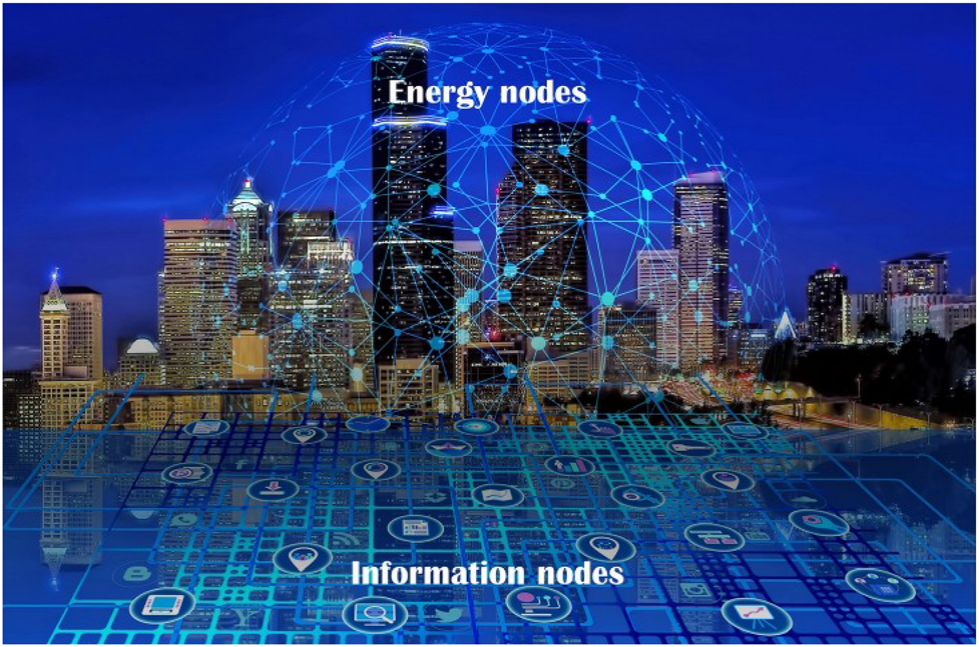
The wireless system of the future smart city.

Metamaterial is an epoch-making and fast-developing research field [1], [2], [3], [4]. It is an artificial structure consisting of sub-wavelength elements with unusual and advantageous properties not existing in nature [5], [6], [7]. Electromagnetic (EM) metamaterial can flexibly manipulate EM waves in unconventional ways and has yielded a variety of exotic phenomena and devices [8], [9], [10], [11]. Vesalago’s theoretical work and Pendry’s practical demonstration have promoted widespread applications of metamaterial [12, 13]. Moreover, metamaterial has also gained consideration in acoustic and visible regions [13], [14], [15], [16], [17], [18], [19], [20]. Recent researches have shown that metamaterial can enhance various properties of antenna, such as bandwidth, gain, size, etc. [21]. Metamaterial has also demonstrated outstanding potential in holography, anomalous reflection, subwavelength focusing, EM metalen, perfect absorber, cloaking, 5G communication, Internet of things (IoT), etc. [22, 23]. Metasurface, a planar EM metamaterial, has shown compelling capabilities for manipulating EM waves [24], [25], [26], [27], [28], [29], [30], [31], [32], [33], [34]. Tens of thousands of low-power, miniaturized smart terminal mobile devices and wireless sensor network nodes (see in Figure 1) are distributed on a large scale in various corners of the world. It is challenging whether these tens of thousands of mobile devices can adapt and continuously work in multiple complex environments, which has motivated significant research interest in wireless power transfer (WPT) and wireless energy harvesting (WEH) to improve transmission performance [35].

Since the end of the 20th century, wireless communication and IoT have made significant progress, enabling high-speed WIT [36, 37]. The continuous improvement of the electronics industry, the rapid iteration of wireless communication technology, and the fast-growing popularity of artificial intelligence (AI) have made modern society more efficient and intelligent [38]. The next-generation wireless technology is the convergence of communication, environmental sensing, and distributed computing [39]. Besides, the number of wireless devices has increased dramatically in the smart city [40, 41]. The wireless devices are considerable heterogeneity nodes with different information and energy requirements [42]. However, we have to endure the inconvenience of more and more chargers and charging cables [43, 44]. Meanwhile, numerous electronic detectors and sensors are installed in hard-to-reach places with the development of modern industry [38, 45].

**Figure 2:**
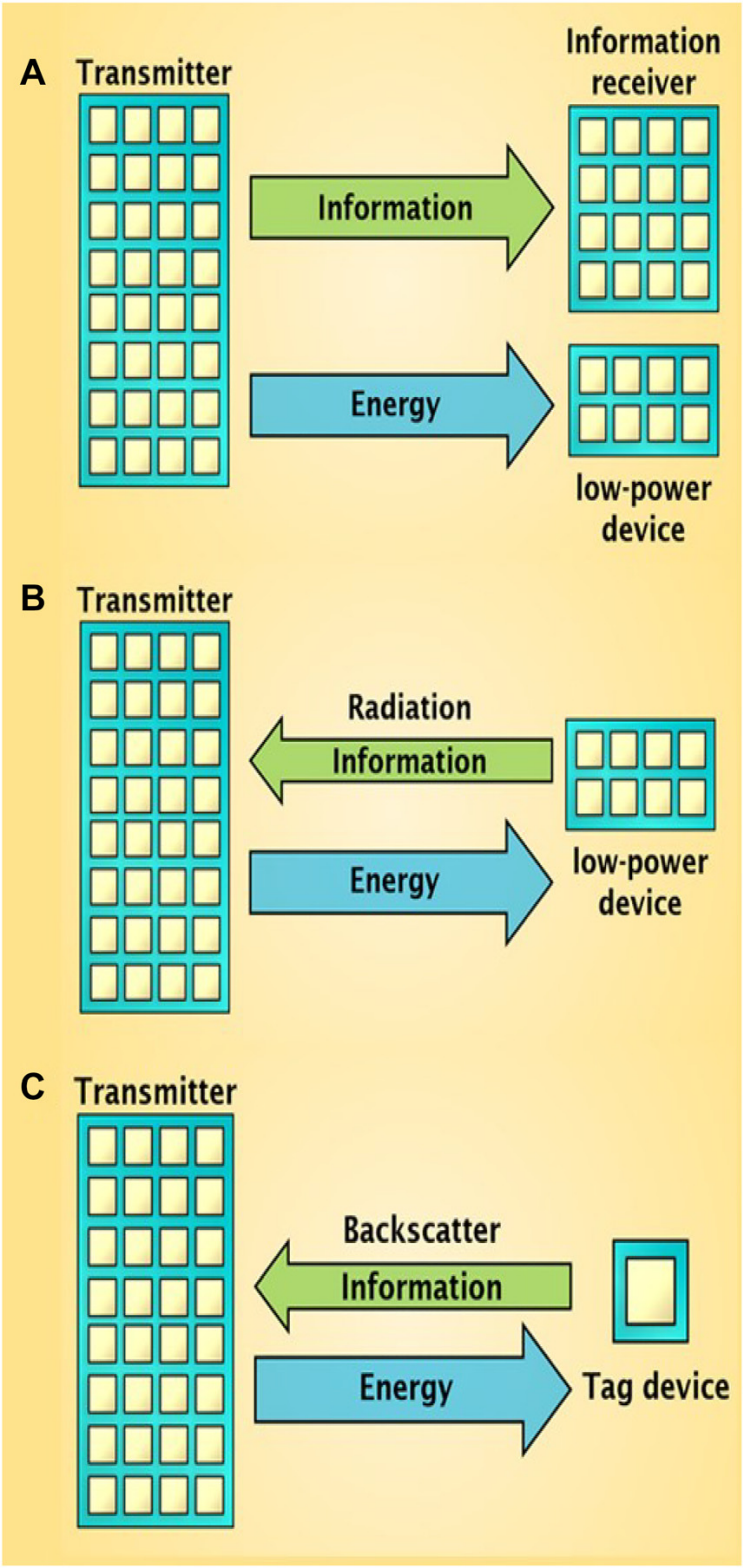
Different SWIPT systems. (A) Wireless power communication system. (B) Wireless power radiation communication system. (C) Wireless power backscatter communication system.

The explosive growth of wireless devices has led to the rapid development of WPT and WEH technologies [46]. These technologies have opened up the possibility of wireless charging [47], [48], [49], [50], [51], [52]. EM waves transmit information in a non-contact way. Essentially, information is a kind of manipulated energy and EM waves are its carrier [53, 54]. EM waves are an untapped energy source and abundant in wireless communication [42, 55]. Although the energy level of EM waves is typically low, they can still charge low-power devices as a Wireless power source [56, 57]. However, for quite a long time, the studies of information and energy were separated. Previous researchers mainly focused on the information characteristic and ignored the energy characteristic of EM waves. Since the beginning of the 20th century, researchers have been looking for an effective wireless power transfer method [43]. Varshney et al. [58] first proposed the concept of simultaneous wireless information and power transmission (SWIPT) in 2008. With the dual physical characteristics of EM waves, SWIPT technology can transmit both information and energy, making it possible for devices to communicate and work simultaneously [59, 60]. Subsequently, many scholars have updated and improved SWIPT technologies. WPT technologies can deliver the energy from a source to a wireless device via free space instead of traditional wires [61, 62]. WPT technologies commonly consist of near-field and far-field transmissions [53, 63, 64]. The near-field WPT utilizes the inductive coupling effect of non-radiative EM fields, while the far-field WPT utilizes the acoustic, optical, and microwave energy carriers. WEH technology can convert radio frequency (RF) energy into direct current (DC), which provides a continuous power supply for low-power devices compared to the current limited-life chemical battery [65], [66], [67]. Currently, the wireless communication system is undergoing tremendous changes. The novel SWIPT technology, including WIT, WPT, and WEH, shows significant implications for the pervasive application of renewable energy in our daily lives [63, 68]. An efficient SWIPT system fundamentally relies on the capabilities of WIT, WPT, and WEH. Such a system requires the consideration of the trade-off between information and energy. In Figure 2, SWIPT systems can be categorized into three different types:

- Wireless power communication system (WPCS): One piece of equipment in WPCS has an information receiver and a low-power device. The low-power device, such as the management unit of the implantable medical devices, may be the control module of wireless equipment. Information and power are simultaneously transmitted to the equipment in the downlink. The data decoded by the information receiver is an instruction to adjust the operational status of the equipment, while the power can provide the required energy of the low-power device. In the future, implantable medical devices may use WPCS to increase service life further.
- Wireless power radiation communication system (WPRCS): One equipment in WPRCS is a low-power device, such as the sensors for detecting various information, the concentration of CO_2_, the temperature, the humidity, the light intensity of the greenhouse, and the PH of the soil [69]. For modern intelligent agriculture, a massive number of sensors need to be deployed. However, these sensors are size constrained with low-capacity batteries. Once the battery is exhausted, the sensors will not work, which reduces the lifetime of the detecting system. Moreover, some sensors may be deployed beneath the soil or water, which is inconvenient to replace batteries. Therefore, the low-power sensors in WPRCS can receive the energy from the transmitter in the downlink so that the low-power device can work typically. Then, the low-power device can use the received power to transmit information back to the transmitter in the uplink. The WPRCS can improve grain production and productivity with real-time information monitoring for modern intelligent agriculture.
- Wireless power backscatter communication system (WPBCS): Backscatter communication reflects the EM wave in a specific modulation to make the receiver decode the backscatter signals. One piece of equipment in WPBCS may be a backscatter tag device, such as radio frequency identification (RFID). Information and energy are transmitted in the uplink and downlink, respectively. EM waves received by a tag device are used for the backscatter modulation to send the information of the tag device to the reader in the uplink. More generally, the tag device can collect ambient EM energy for scattering communication when the ambient EM energy is suitable and sufficient.

This review elaborates on current major research topics and discusses future development trends of metamaterials in WIT, WPT, WEH, and SWIPT technologies and their future trends. It is organized as follows. Section 2 briefly explains the WIT technologies. Metamaterials are affecting the basic architecture of wireless communication systems. Recent advances in metamaterials for WPT and WEH are presented in Section 3. Different schemes can improve the energy distribution, increase transmission distance and efficiency of the WPT and WEH system. The part of metamaterials in SWIPT is given in Section 4. We summary different approaches to explore new energy channels to meet complex SWIPT system needs. Finally, this review is concluded with perspectives of these rapidly growing SWIPT technologies.

## 2 Metamaterials for WIT

**Figure 3:**
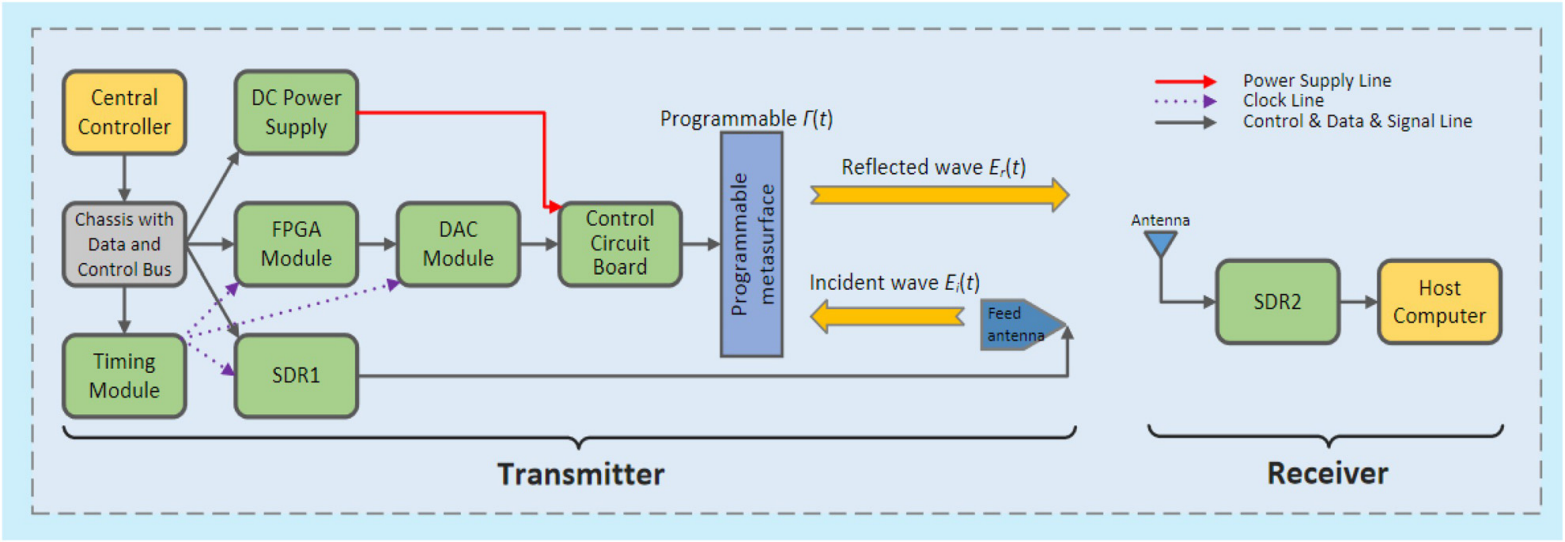
The hardware architecture of the programmable metasurface-based wireless communication system [70]. Reprinted from [70], with the permission of IEEE Publishing.

The programmable metasurface is composed of programmable units, each of which has tunable spatial phase-shift functionality by using positive–intrinsic–negative (PIN) diodes. It can achieve programmable controls of EM waves in real-time by integrating with the field-programmable gate array (FPGA). Programmable metasurfaces are potential in wireless multiplexing techniques because they can encode and transmit information without using traditional antennas or mixer components, as shown in Figure 3. On the other hand, the digital coding strategy bridges the physical and digital worlds, making the metamaterial realize direct information processing. Metamaterials have shown great potential to bring revolutionary WIT applications with outstanding advantages. In ref. [70], a programmable metasurface was proposed to realize single carrier quadrature phase-shift keying (QPSK) WIT. By exploiting the dynamically controllable property of the programmable metasurface, the phase of the reflected EM wave is directly manipulated in real-time according to the baseband control signal. Programmable metasurface does not require any filter, mixer, or wideband power amplifier as a low-cost transmitter for wireless communications. The experiments revealed that the metasurface-based wireless communication system could achieve comparable performance with less hardware complexity and thus leading to a new architecture with great potential for the future wireless communication system.

**Figure 4:**
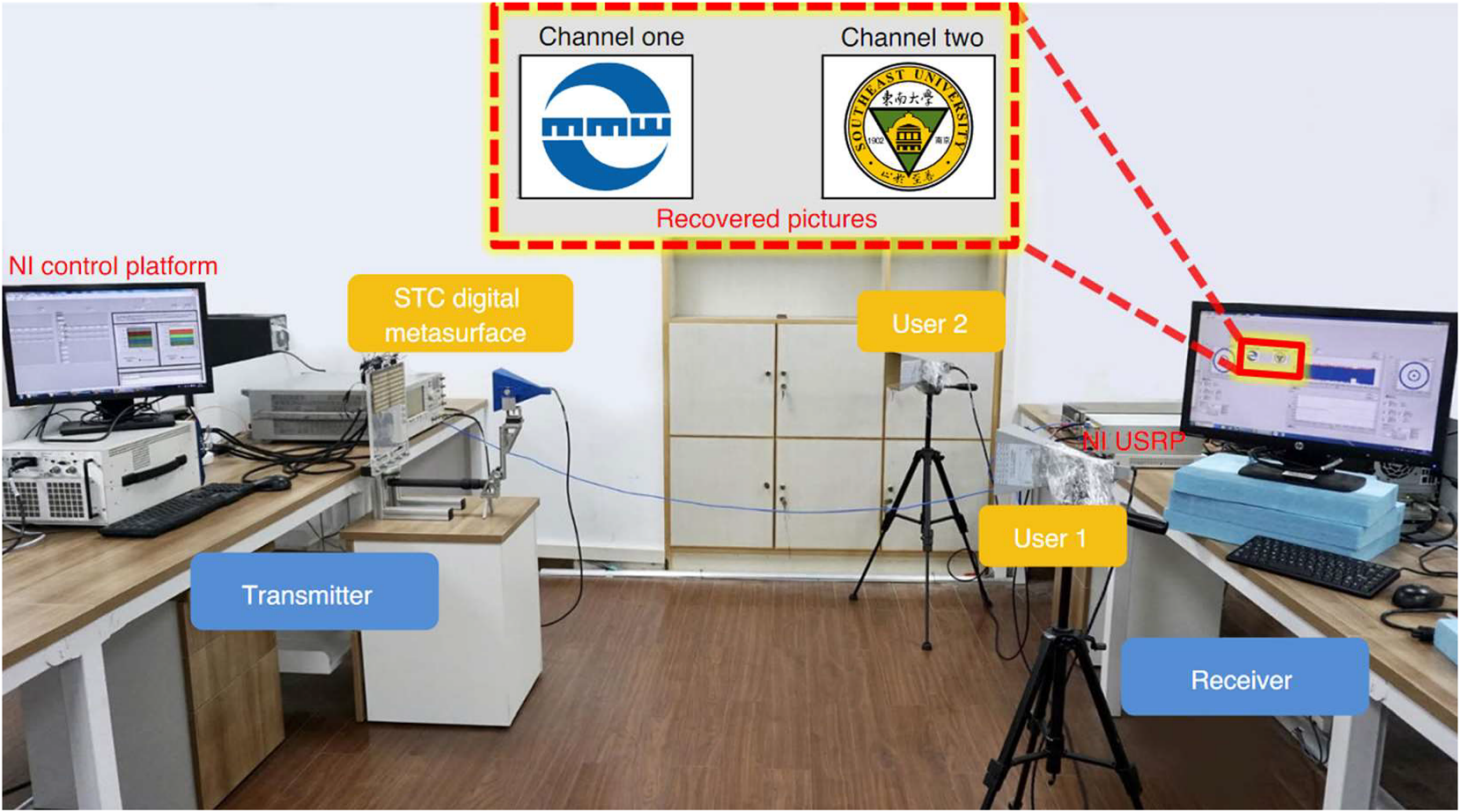
The wireless information transmission with digital metasurfaces [71]. Reprinted from [71], with the permission of Springer Nature Publishing. © 2021, The Author(s), under exclusive license to Springer Nature Limited.

**Figure 5:**
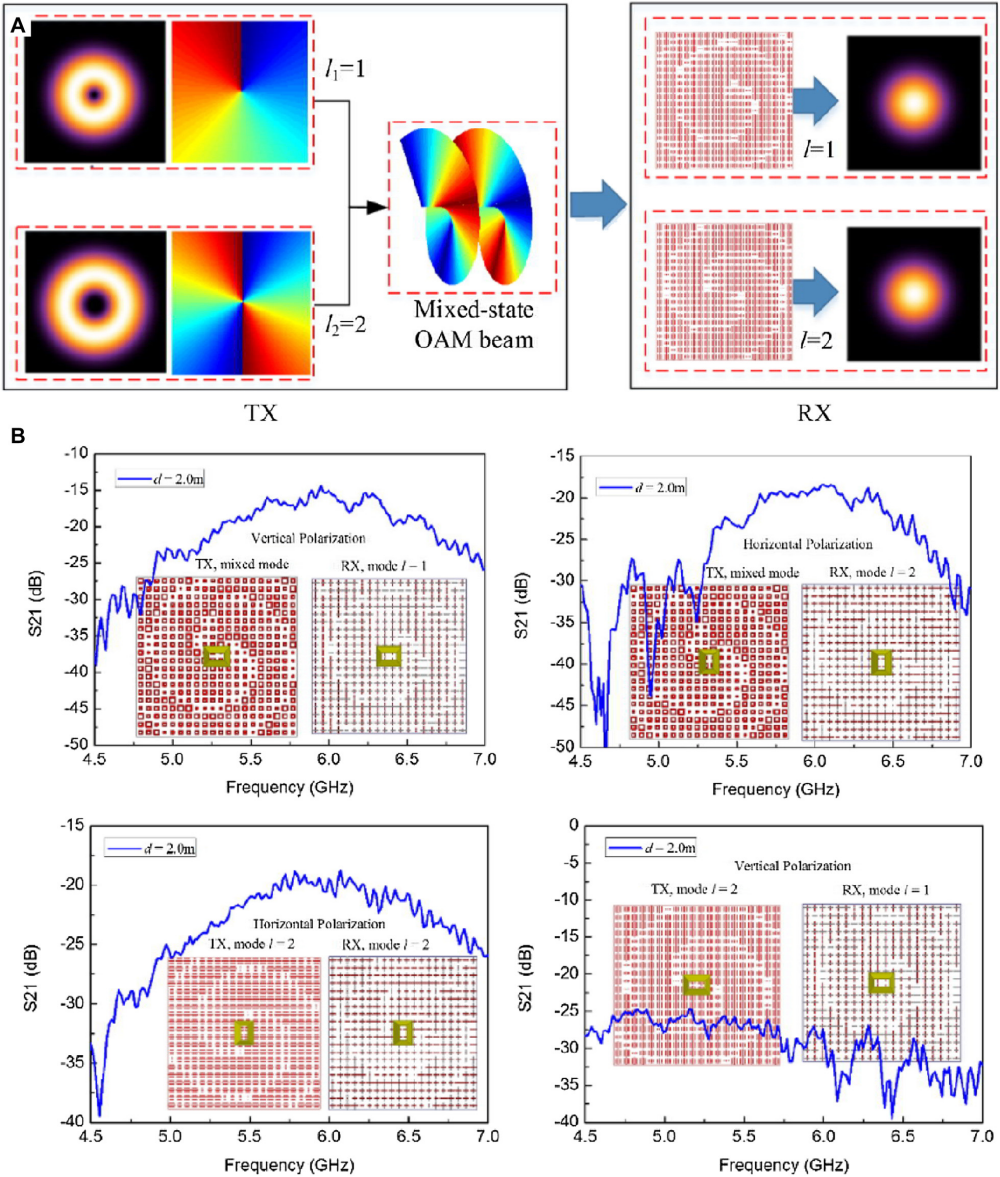
Metasufaces for information demodulation. (A) Metasurfaces for reception and separation of the information of the mixed-mode vortex waves. (B) Different metasurfaces for separation of the OAM modes [72]. Reprinted from [72], with the permission of Optical Society of America Publishing.

Zhang et al. [71] reported a wireless communication system with a digital metasurface in Figure 4. The power intensities transmitted can be controlled independently and simultaneously at the specific harmonic frequency for each user by changing the 2 bit space-time matrices. Arbitrary user locations can be realized by using the optimization process when the dimension of the matrices is sufficiently large. By encoding space-time matrices through multiple channels, information can be directly transmitted to multi-user at different locations simultaneously, without digital-to-analog conversion and mixing processes. The dual-channel wireless communication system can send two different pictures to two users simultaneously in real-time. The digital metasurfaces can implement secure and low-cost space- and frequency-division multiplexing in a dual-channel wireless communication system. Metamaterials can also be used for information demodulation. Yu et al. [72] proposed metasurfaces transmitting and receiving a mixed-mode orbital angular momentum (OAM) vortex wave, as shown in Figure 5. Experimental results show that the information of the mixed-mode OAM vortex waves can be detected, received, and separated by pure OAM-mode transmitting and receiving metasurfaces. With the reciprocity, they can demodulate the mode information of OAM vortex waves, which provides an effective way for information reception and demodulation.

In this section, we mainly review the application of metasurfaces in the field of WIT. Metasurfaces are of particular interest because of their low loss, ultrathin thickness and easy integration. They have been applied to encode and decode information in the wireless communication system. Unlike the conventional antenna, the metasurfaces can control the EM waves for a variety of functions with its own adjustable characteristics. Furthermore, digital coding metasurfaces combine information science and digital signal processing, leading to the concept of information metamaterials and metasurfaces. The advantages of metamaterials for WIT are summarized in Table 1.

**Table 1:** The advantages of metamaterials for WIT.

Type of metamaterials	Advantage
Programmable metasurface	Low loss, ultrathin thickness and easy integration
	Integrated with PIN diodes and varactors
	Dynamically control the EM waves among many different functions in real time
	Encode and transmit information without using traditional mixing processes
	Combine information science and digital signal processing

## 3 Metamaterials for WPT and WEH

The wireless communication system requires higher information transmission and processing requirements, including faster information rates, lower latency, wider bandwidth, broader coverage, and environmental friendliness. Therefore, the number of wireless devices will increase rapidly in the future wireless communication system. Energy supply for these wireless devices has become a challenging issue. Limited by energy density and transmission distance, WEH is still in infancy. WEH technology enables wireless devices to use the received EM waves to charge themselves while communicating. In this way, EM waves can be seen as an intermittent energy source that enables tens of thousands of wireless devices to communicate and energy themselves anywhere, anytime, and on the move. This section reviews and discusses the recent advances in metamaterials for WPT and WEH.

### 3.1 Metamaterials for WPT

In recent years, the manipulation of EM waves has attracted great interest. The EM beam can be manipulated in many novel ways with metamaterials, such as focusing beam, non-diffractive beam, etc. These beams provide alternative solutions for the SWIPT system because they provide powerful help for energy aggregation and transmission. Focusing beams can concentrate energy to a specific area, and the energy density will be significantly increased. Non-diffractive beams can increase energy transmission distance, which is helpful for long-distance communication and energy supply.

#### 3.1.1 Focusing beam metamaterials

**Figure 6:**
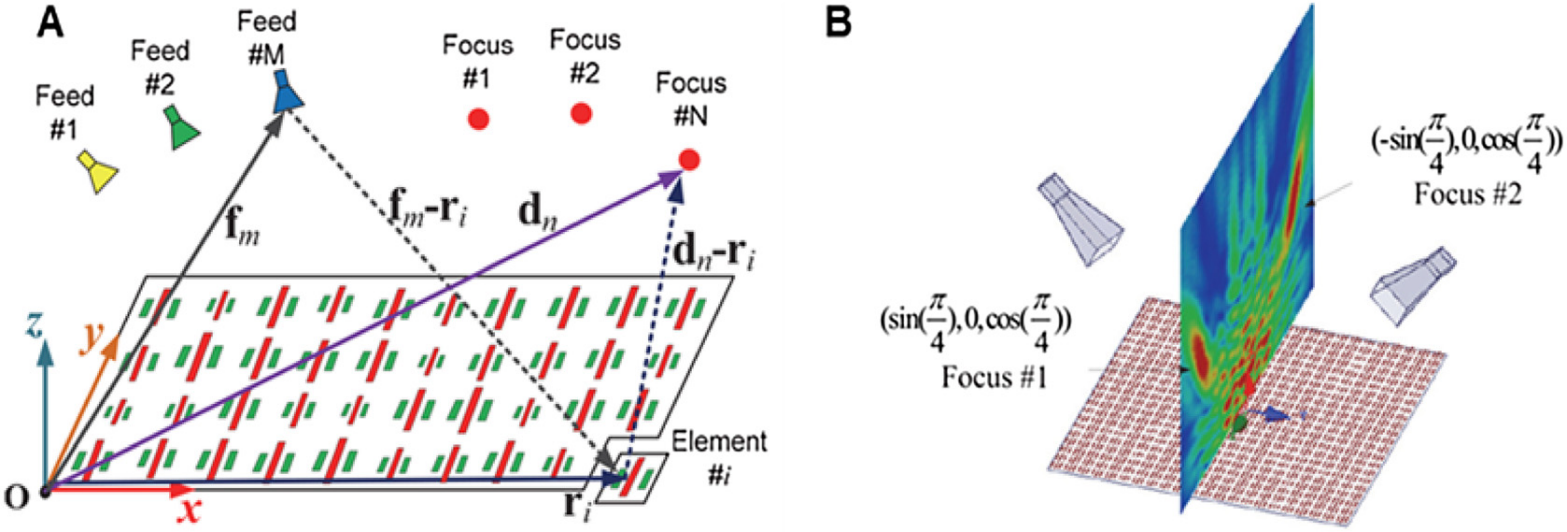
NFF metasurface. (A) The reflective metasurface for the multi-feed and multi-focus WPT system. (B) Full-wave simulation of the reflective metasurface [73]. Reprinted from [73], with the permission of IEEE Publishing.

**Figure 7:**
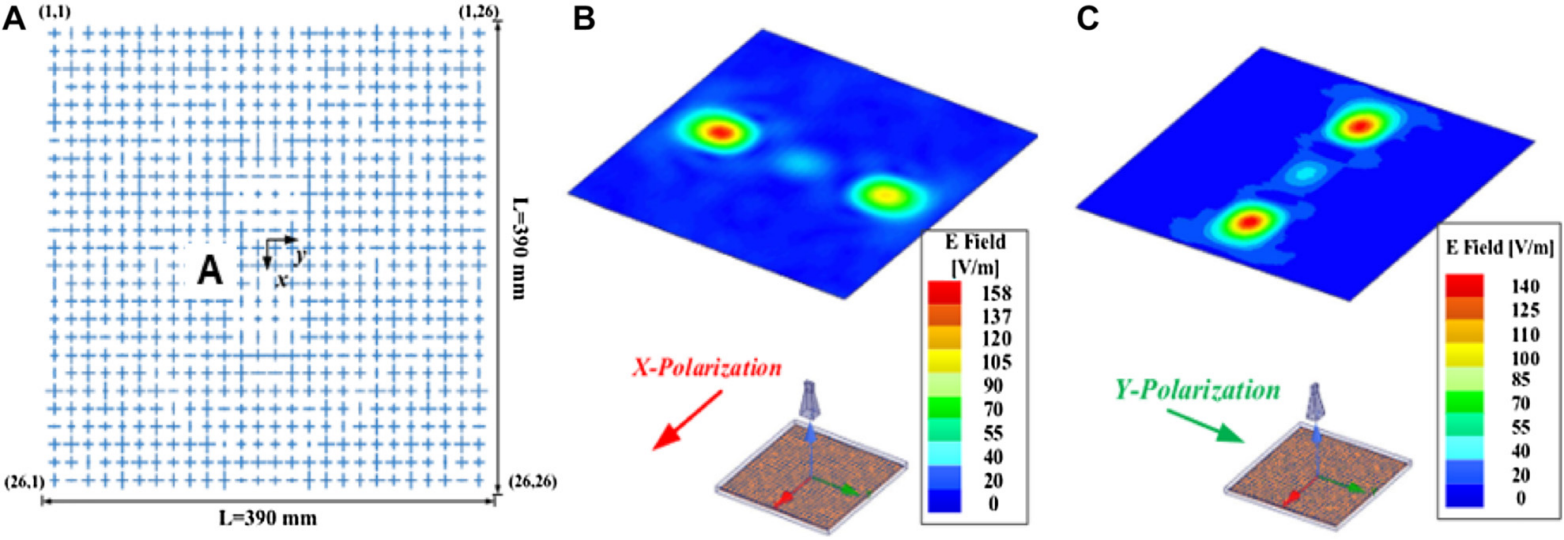
NFF metasurface for WPT. (A) The geometry of single-feed, dual-focus reflective metasurface. (B) and (C) Full-wave simulation of the multi-focus metasurface [74]. Reprinted from [74], with the permission of IEEE Publishing.

Focusing beam metamaterials can increase the energy strength in a convergent manner so that wireless charging devices can collect more EM energy to maintain a longer working life. It was theoretically proven that an excellent focus performance in the near-field region would be achieved with proper phase regulation of metamaterials. Near-field focus (NFF) transmission technology has been extensively applied in many aspects, such as medical ultrasound treatment, optical imaging, non-contact sensing, and microwave hyperthermia [75]. Yu et al. presented a novel NFF metasurface with multi-focus characteristics for high-efficiency WPT [73]. The NFF metasurface is shown in Figure 6. Full-wave simulation and experiment results demonstrate that the proposed NFF metasurface can be applied in many fields, including multi-user WPT systems, remote sensing devices, biological wearable devices, and multi-object RFID systems. Zhang et al. present a new multi-focus NFF reflective metasurface for WPT in Figure 7 [74]. In terms of multi-beam phase synthesis of the reflective metasurface, the multi-focus NFF metasurface with independent modulation characteristics can realize multi-focus and high-efficiency WPT. Through full-wave simulation and experiment results of three cases, single focus, dual-focus, and single focus with dual-polarization can respectively realize the maximum focusing efficiency of 71.6%, 68.3%, and 65.9%, which demonstrates the stability and feasibility of the multi-focus NFF reflective metasurface for WPT applications.

**Figure 8:**
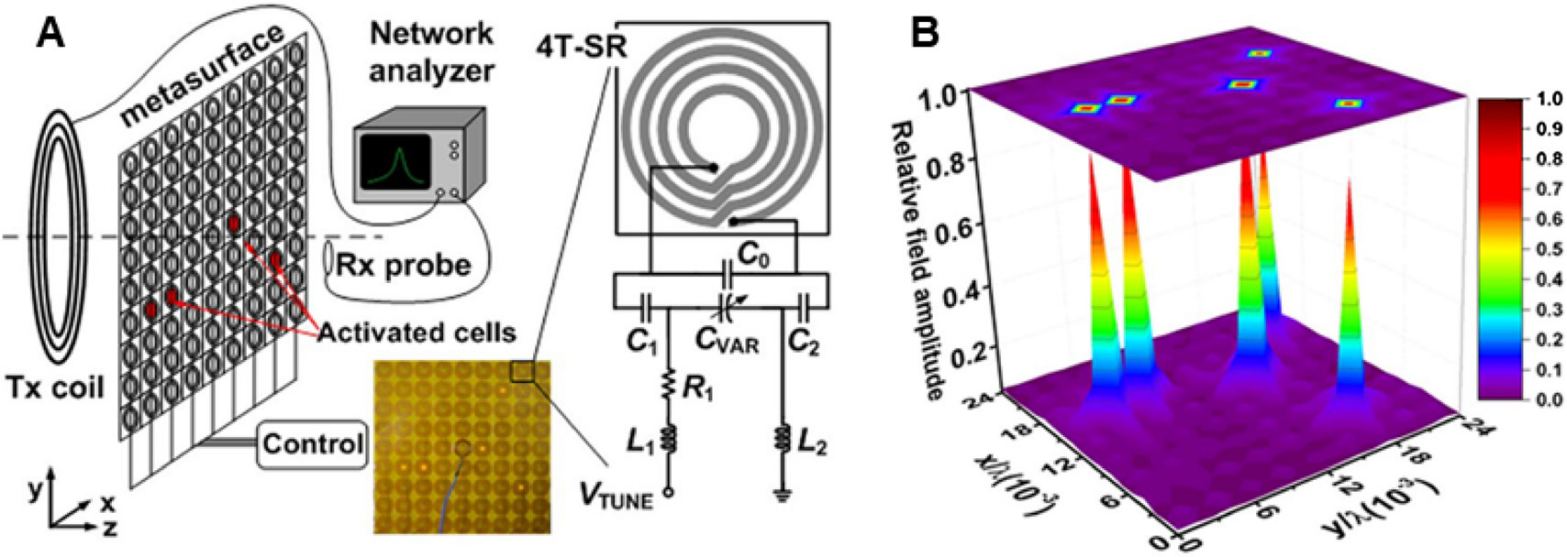
Active NFF metasurface. (A) A schematic of the active metasurface for focusing beam. (B) Full-wave simulation of the active metasurface [22]. Reprinted from [22], with the permission of Springer Nature Publishing. © 2019, The Author(s).

Yang et al. [22] introduced an active focusing metasurface for field-localizing WPT in Figure 8. The active metasurface was proposed with multi-functionality due to its frequency switching and tuning capability. The focus location, shape, and intensity can be manipulated as desired with the proposed active focusing metasurface, which overcomes the limitations of passive metamaterials and effectively enhances energy transmission to the intended devices while reducing leakage to unwanted areas. The active dynamic reconfigurable capability provides a wide range of versatile applications requiring precise energy transmission control.

**Figure 9:**
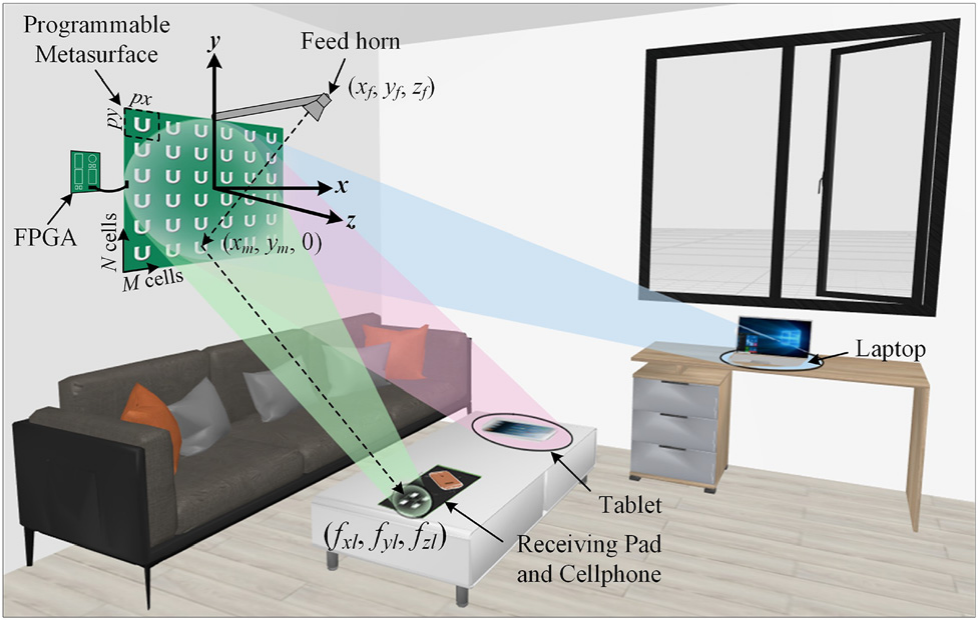
Adaptively intelligent wireless charging scenario using NFF programmable metasurface [76]. Reprinted from [77], with the permission of IEEE Publishing.

An adaptively intelligent WPT system is proposed by using a 2 bit programmable metasurface in Figure 9, in which an in-door dynamic charging application scenario is described and the NFF technique is adopted to transmit wireless powers [76]. Han et al. investigated the digital phase-shift quantization of PMS elements to implement dynamic WPT. The results indicate that a 2 bit quantization scheme is a preferred solution. A 2 bit 12 × 12 PMS operating at 5.8 GHz is designed, fabricated, and tested. High-power focal spots that can be regulated to focus at different near-field positions are measured in an anechoic chamber. Experimental results show that the proposed 2 bit PMS can continuously transmit the wireless powers to the receiver even though the target is moving, and the WPT efficiency is maintained over 9.1%, which has about 16.3 dB improvement at the end of the trajectory compared to the fixed NFF beam. This work firstly examines the possibility of the dynamic WPT using PMS strategy, offering a brand-new manner for the adaptively intelligent WPT systems.

#### 3.1.2 Non-diffractive beam metamaterials

**Figure 10:**
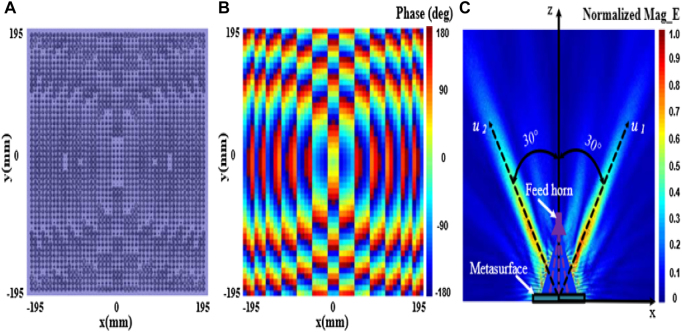
Non-diffractive beam metasurface. (A) Layout of the metasurface. (B) The compensation phase distribution of the designed metasurface. (C) The normalized E-field amplitude distribution of the dual pseudo-Bessel beams on the xoz-plane [78]. Reprinted from [78], with the permission of MDPI Publishing.

SWIPT can realize wireless information and energy transmission, which provides an irreplaceable role in developing a diverse communication system. However, the divergence characteristic of EM wave radiation limits its application. Non-diffractive beam shows the potential to solve this problem. It is more convergent than the conventional beam. As the energy in the non-diffractive area has the characteristics of uniform and high efficiency, it is suitable for WPT. Metamaterials can also be used to generate non-diffractive beams. It is not easy to maintain high efficiency and high directionality when the beam is deflected. The performance of the single off-axis pseudo-Bessel beam is not satisfactory, and multiple pseudo-Bessel beams are more challenging to be achieved. In ref. [78], a reflective metasurface is proposed to generate multiple pseudo-Bessel beams with accurately controllable directions. From Figure 10, it can be seen that the dual pseudo-Bessel beams have been generated by the metasurface simultaneously, and the propagation directions of them meet the predetermined settings. The radiation power is concentrated along the propagation axes. To further prove this, it can be observed that the amplitudes of the normalized E-field are perpendicular to the two propagation directions. Kou et al. [79] proposed a multilayer amplitude-phase-modulated metasurface to generate a pseudo-non-diffractive high-order Bessel beam. The multilayer amplitude-phase-modulated metasurface can transform a quasi-spherical wave emitted from the feeding source into a pseudo non-diffractive high-order Bessel vortex beam. Full-wave simulation and measurement results confirm that Bessel vortex beams can be effectively generated using the proposed amplitude-phase-modulated metasurface. These proposed methods and metasurface for generating Bessel beams can be applied to multi-user WPT, wireless communication, and multi-object RFID systems with a considerable distance [80].

With exciting quasi non-diffraction, self-bending, and self-healing properties, Airy beams can be applied in WPT and wireless communication systems with a considerable operating distance. In ref. [81], an Airy beam is experimentally generated by a metasurface composed of a single-layer of square C-shaped complementary split-ring resonators. The magnitude and phase of each metasurface element can be controlled by modifying its rotation angle to shape the desired Airy beam. Airy beams exhibit intriguing properties such as non-spreading, self-bending, and self-healing and have attracted considerable recent interest because of their many potential applications in photonics, such as beam focusing, light-sheet microscopy, and biomedical imaging. In ref. [82], a metasurface composed of silicon posts was designed and fabricated to generate an Airy beam at THz band. The generated achromatic Airy-beam-based metalens exhibits self-healing properties that are immune to scattering by particles and that it also possesses a more enormous depth of focus than traditional metalens. Such technology can push the boundaries of long-distance SWIPT in the optical field.

#### 3.1.3 Relay-enhanced metamaterials

The coupling between the transmitter and the receiver can be enhanced by focusing the near-field with the aid of metamaterial insertion [49, 83], [84], [85], [86], [87], [88], [89]. For coupling WPT systems, energy is transmitted between resonators via coupling of evanescent fields. Thus, the unique evanescent wave amplification property of metamaterials can be applied in this case [90], [91], [92], [93]. Recently, various metamaterials have been theoretically and experimentally verified to improve the WPT efficiency. Since most WPT systems are based on magnetic near-field coupling, it is sufficient to use negative permeability metamaterials. Wang et al. [94] experimentally implemented a WPT scheme using negative permeability metamaterials. As a pioneering work of metamaterials in the WPT field, it had a meaningful influence on the following researches on different metamaterial designs and implementations. Recently, a new intermediate component, so-called near-field plates, has been proposed to suppress the backward radiation generated by the transmitting coil. The near-field plate helps suppress the backward radiation and thus forms a unidirectional near-field pattern.

**Figure 11:**
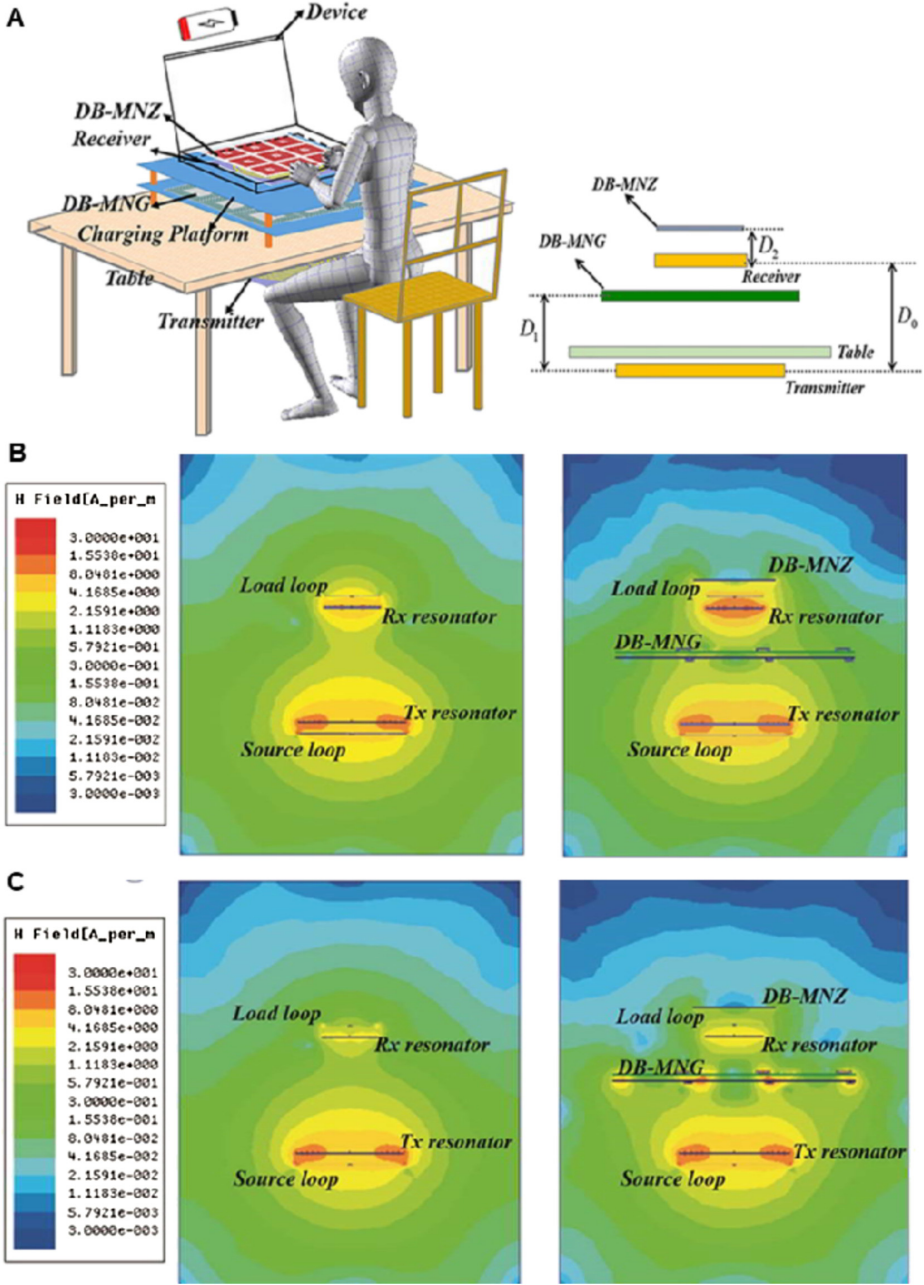
Relay-enhanced metamaterials for coupling WPT. (A) WPT system integrating with the dual-frequency negative permeability and near-zero permeability metamaterials. (B) and (C) Magnetic field distribution at 13.56 and 27.12 MHz, respectively [77]. Reprinted from [77], with the permission of IEEE Publishing.

Lu et al. [77] reported a novel asymmetric WPT system for enhancing efficiency and reducing leakage magnetic field by integrating with the dual-frequency negative permeability and near-zero permeability metamaterials. Figure 11(b) shows the results for the four cases of the WPT system at 13.56 and 27.12 MHz. The results show that strong magnetic fields appear around the receiver in the situation of the WPT system with dual-frequency negative permeability metamaterial. The simulation proved that the dual-frequency negative permeability metamaterial could increase the magnetic field around the receiver at two frequencies. The dual-frequency near-zero permeability metamaterial can shield the magnetic field generated by the operating and second frequencies while allowing other areas to pass Wi-Fi signals.

**Figure 12:**
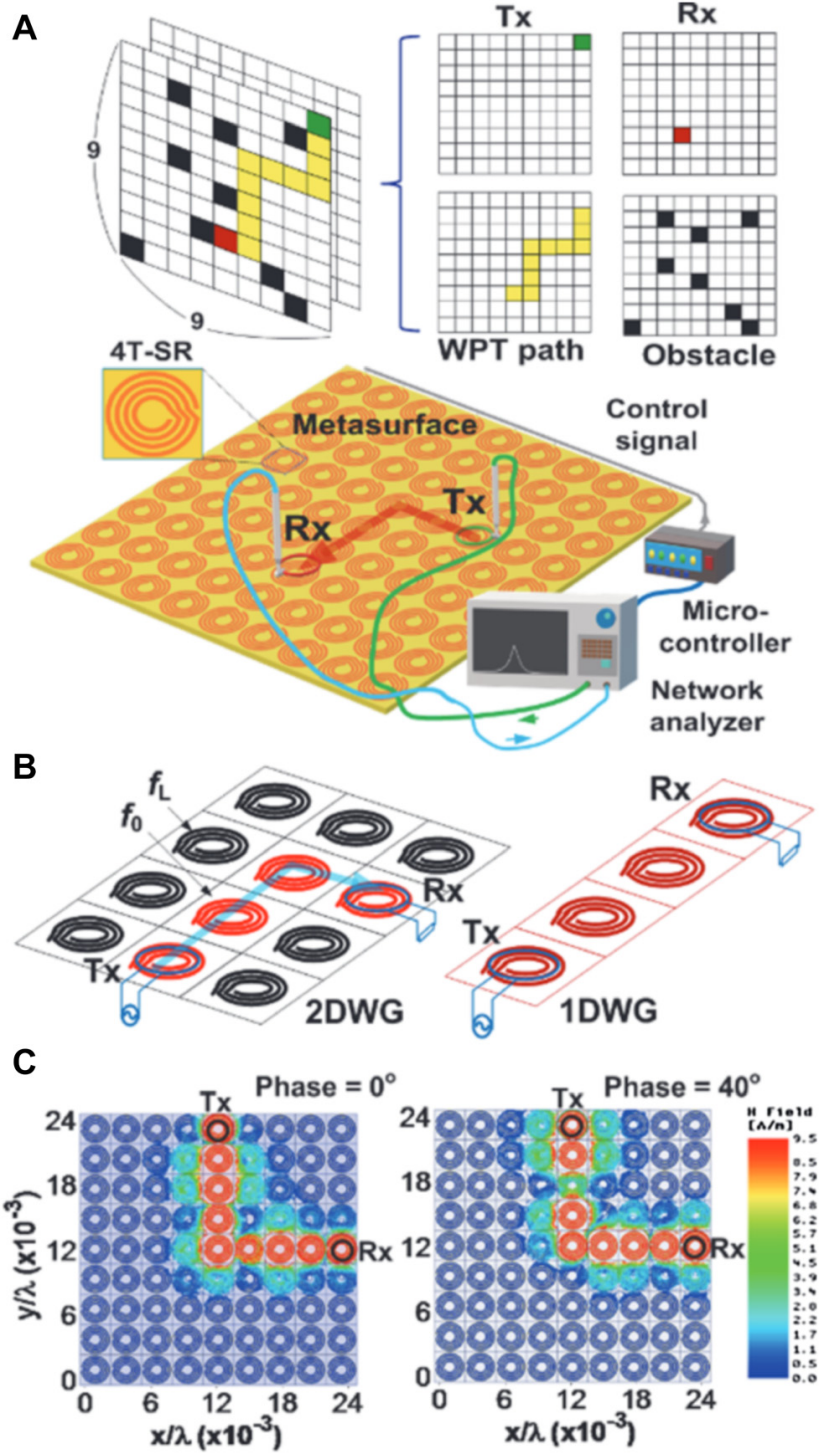
Tunable metamaterials for coupling WPT. (A) Tunable metamaterial for the power transmission. (B) Energy transmission path. (C) Distribution of the EM energy [95]. Reprinted from [95], with the permission of IEEE Publishing.

For applications of WPT technologies, the limitation of the energy transmission distance and energy leakage to the undesired directions are two critical problems. Thus, different approaches to energy transmission route control are required. One of the fundamental approaches to enlarge the transmission distance is to apply an intermediate coil between transmitting and receiving coils. It has been proven that the WPT efficiency can be significantly improved using such middle resonant coils. The concept of medium coils opened possibilities for controlling the energy transmission route. The desired power transmission route can be defined by adequately designing a topology structure with identical coil resonators. Desired energy transmission path can be created by distributing a series of identical WPT resonators along a specific route. Bui et al. [95] investigated a deep neural network (DNN)-based design of the tunable metamaterial for WPT, as shown in Figure 12. When transmitting 10 W RF power, a peak efficiency of 56.8% can be achieved. The DNNs were trained using 23,070 randomly selected samples. For predicting the spectra, an accumulated MSE less than 1.5 × 10^−3^ is achieved for 97.3% of the 1929 test set. The results show that the DNN provides an alternative and efficient design method for the metamaterial.

**Figure 13:**
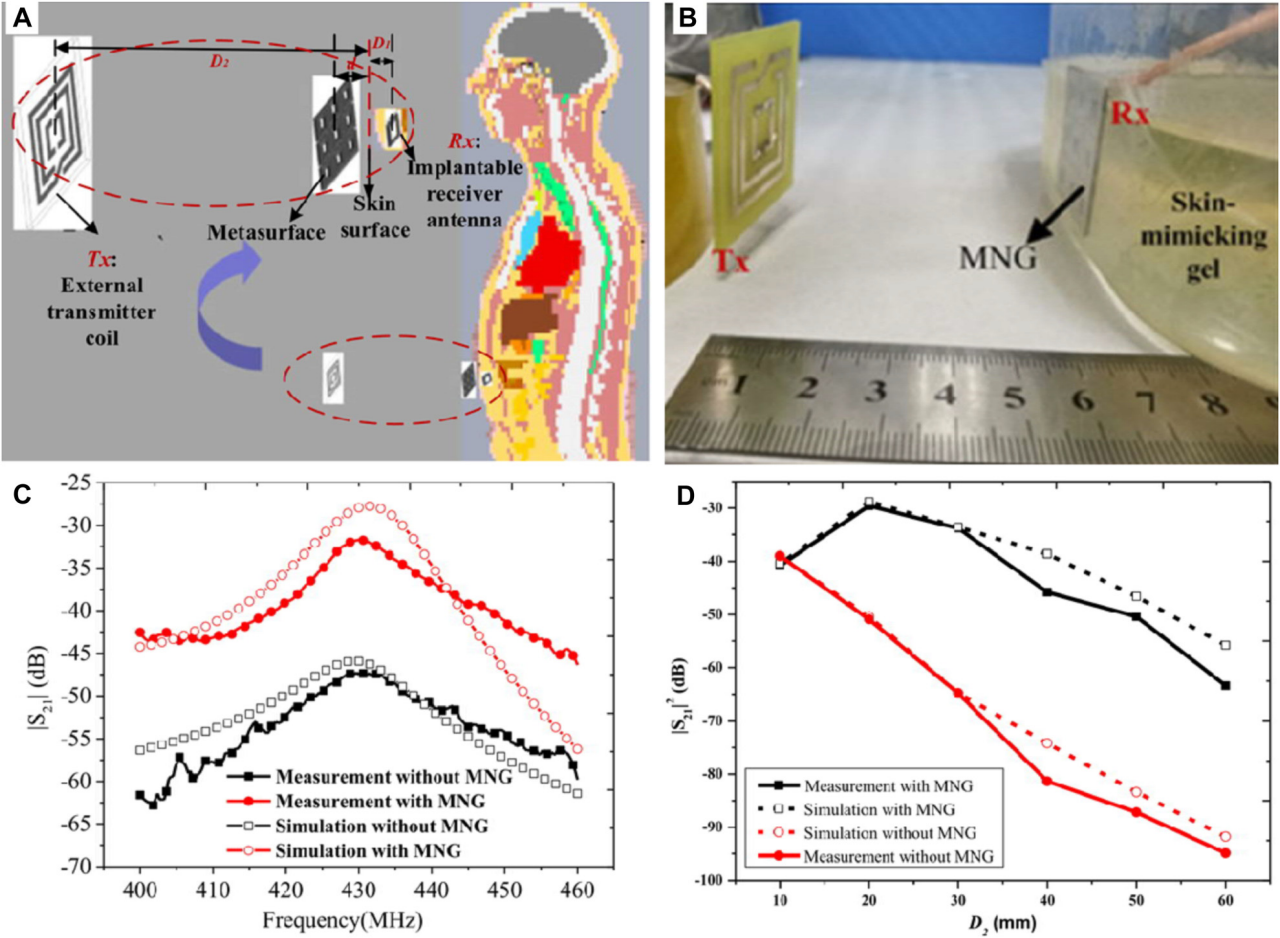
Implantable metamaterials for coupling WPT. (A) WPT system integrates with negative permeability metasurface as a wearable device. (B) The testing scenarios. (C) and (D) The comparisons of measured results with and without negative permeability metasurface [96]. Reprinted from [96], with the permission of IEEE Publishing.

In the past decades, tremendous progress in the technological world has brought revolutionary changes to the medical field [98]. A variety of implantable medical devices, such as blood glucose monitors, biomedical sensors, gastric stimulators, intraocular pressure monitoring systems, and implantable pacemakers, are used for patient treatment and health monitoring [99]. Generally, most implantable medical devices require surgery to replace the battery, posing a risk to the patient. WPT is a crucial technology applied in biomedical devices [100]. As an alternative, wireless power transmission can avoid surgery and ensure patient safety. Therefore, the importance of wireless power transmission in the field of biomedicine has become increasingly prominent. Li et al. [96] presented a novel implantable magnetic coupling resonate WPT system in Figure 13. It can be seen from the results in Figure 13(c) that the coupling strength has increased by 15.7 dB with or without negative permeability metasurface. Figure 13(d) illustrates that the performance of the WPT system can be significantly improved, especially in the case of a considerable transmission distance. The negative permeability metasurface can be regarded as a wearable device combined with some clothes or medical tape in practical applications.

**Figure 14:**
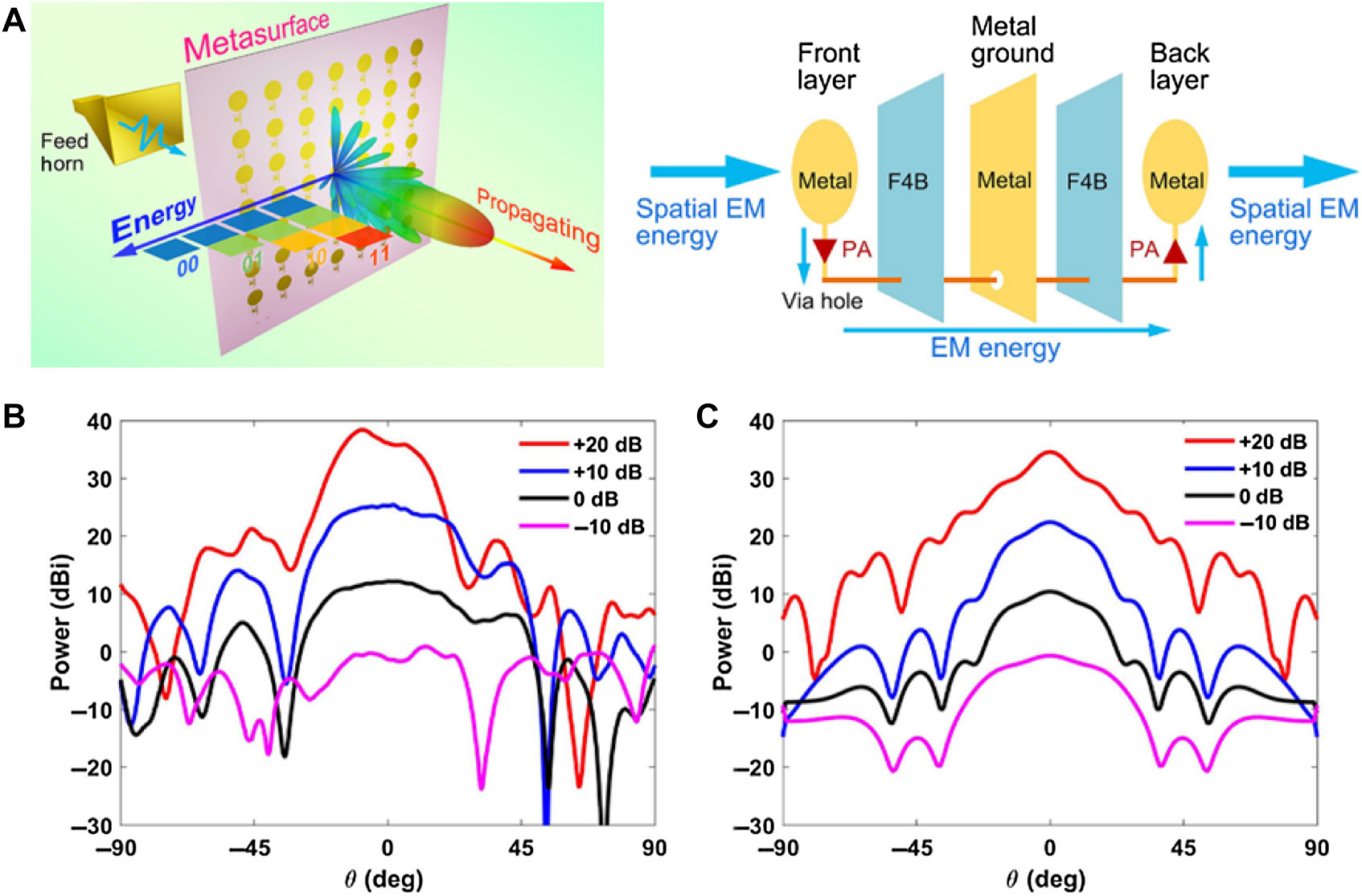
Digital-coding amplifying metasurface. (A) The digital-coding metasurface with active amplifiers. (B) The simulated and measured far-field results of the designed metasurface with different amplification levels [97]. Reprinted from [97], with the permission of American Physical Society Publishing.

Most previous passive and active metasurfaces are almost energy lost when controlling EM waves. Then, the amplifying metasurfaces have been proposed. In ref. [97], a spatial-energy digital-coding metasurface is presented with active amplifiers to realize arbitrary editing of the energy of spatial propagating waves, as shown in Figure 14. Based on the proposed metasurface, the spatial energy of a linearly polarized propagating wave can be amplified and edited into arbitrary magnitudes (including amplification and reduction) by controlling the voltage. A metasurface element integrated with an amplifier chip is designed for long-distance energy modulations in the microwave frequency range.

### 3.2 Metamaterials for WEH

#### 3.2.1 Rectifying metamaterials

Applications of metamaterial-based WEH have attracted numerous attentions. WEH is preoccupied with gathering ambient EM energy and focuses on designing the appropriate energy convert devices. Simplicity and higher energy conversion efficiency are essential factors to be considered to enhance the overall performance of WEH. One of the attractive features of metamaterials is their ability to realize the spatial processing of energy beams without amplifiers, thereby enabling highly efficient WEH. Compared with array antennas, rectifying metamaterial has less stringent requirements on the spacing of the constituent elements, resulting in a denser configuration and a smaller overall footprint [102]. In addition, it has greater structural flexibility, which can match impedance without additional circuits.

When the impedance of the loads and the impedance at the antenna output match, maximum energy transmission can be achieved. The matching network’s responsibility is to increase the rectifier’s input voltage and minimize the transmission loss from the antenna to the rectifier. However, the realization of the array antenna usually requires additional matching circuits and energy combining networks, which leads to complex designs, higher energy losses, and higher costs. The primary function of the rectifier is to convert captured EM waves to DC. The diode determines EM to DC conversion efficiency of the rectifier. Therefore, the diode is recognized as the main component of the rectifier circuit. Simplicity and higher DC efficiency are essential factors to enhance the overall performance of a rectenna system for WEH.

**Figure 15:**
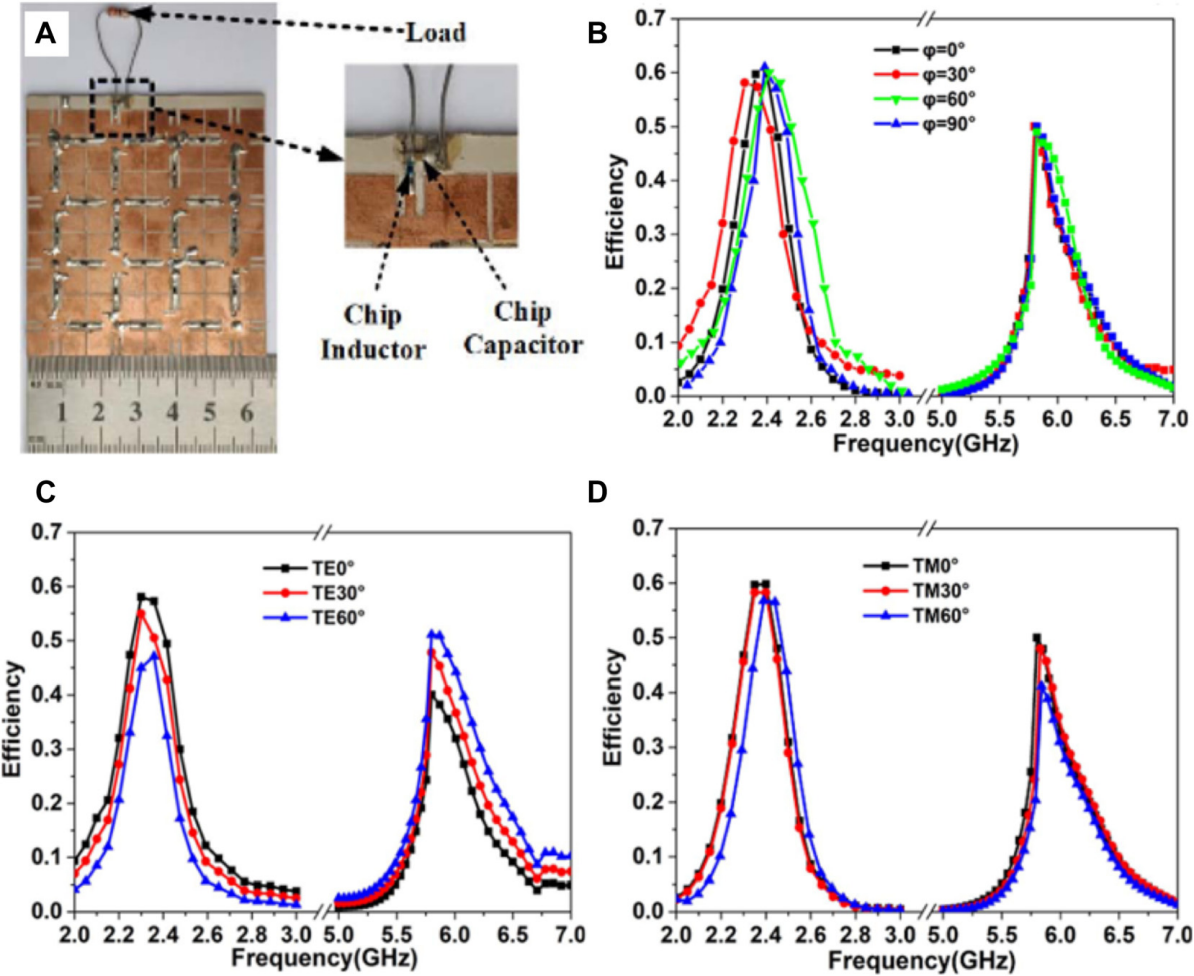
Rectifying metasurface (RMS) for WEH. (A) Dual-frequency and polarization-angle-independent rectifying metasurface. (B)-(D) The measured efficiency for different polarization angles under normal incidence, TE-polarized, TM-polarized oblique incidence, respectively [101]. Reprinted from [101], with the permission of IEEE Publishing.

In Figure 15, Li et al. [101] proposed a dual-frequency and polarization-angle-independent rectifying metasurface for the high conversion efficiency of WEH. The proposed rectifying metasurface consists of one periodic array with integrated diodes, DC feed, and load. The matching network can be directly eliminated due to the metasurface multi-mode resonance and adjustable high-impedance characteristics. In addition, the proposed rectifying metasurface can effectively capture incoming waves with arbitrary polarizations and a wide incident angle range of 60°. The result shows that it can achieve maximum efficiency of 58% at 2.4 GHz and 50% at 5.8 GHz with 0 dBm input energy under different polarizations and incident angles. This work reaches small size, low cost, wide receiving angle, etc., which is very suitable for the WPT of miniaturized IoT terminal devices.

In ref. [103], a rectifying metasurface was designed with an 8 × 8 array with a 2 × 2 rectifying unit while the other cells were terminated with the optimal load resistance. The diode is mounted right at the feed of the metasurface cell, avoiding using a matching network between the metasurface cell and the diode. The output DC energy of the 2 × 2 rectifying unit was connected in both series and parallel to study the ability of the rectifying metasurface to configure the total output current and voltage. The numerical and experimental results show that the proposed metasurface can capture the incident EM waves with radiation to DC conversion efficiencies exceeding 80%.

#### 3.2.2 Hybrid harvesting metamaterials

**Figure 16:**
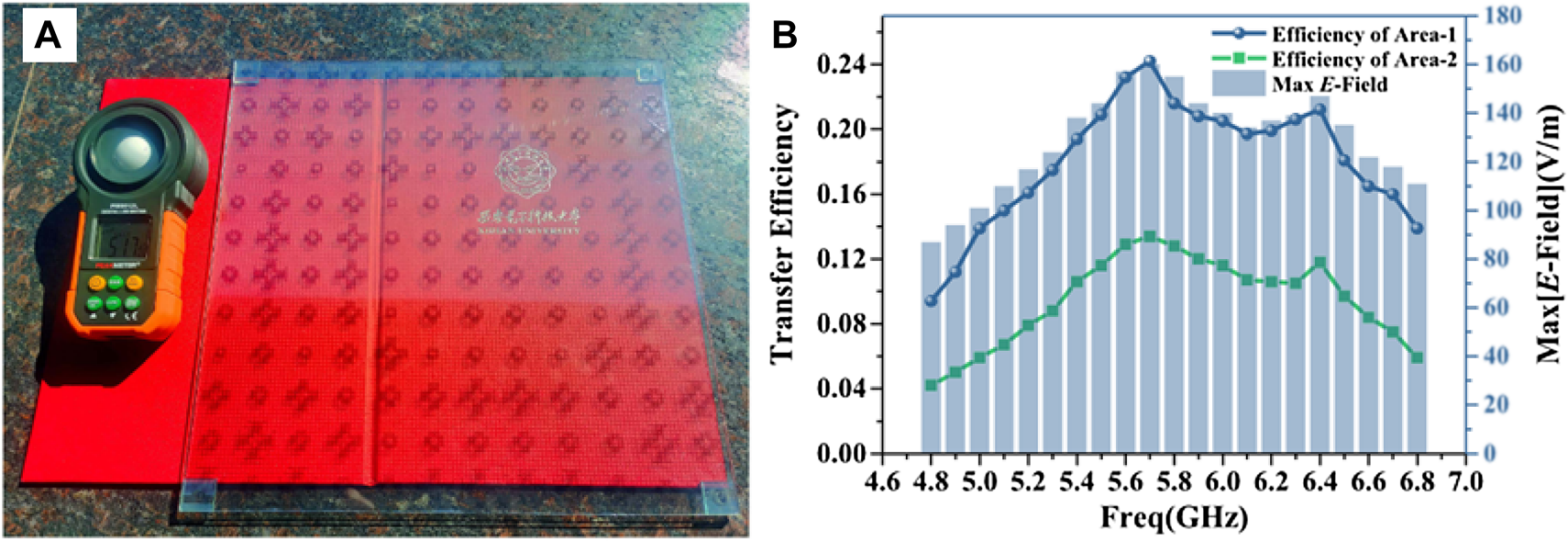
NFF transparent metasurface. (A) Transparent metasurface. (B) The NNF transmission efficiency [104]. Reprinted from [104], with the permission of IEEE Publishing.

In the future SWIPT system, hybrid energy harvesting can enhance robustness. As a candidate, the transparent metamaterial becomes more attractive. With the advancement of micromesh technology, several transparent antennas have been implemented with a balanced performance both electrically and optically. Indium tin oxide (ITO) is generally considered the best compromise between light transmittance and conductivity among these materials. By combining transparent materials with solar panels, hybrid energy collection can be achieved. The use of transparent materials enables hybrid energy harvesting. In Figure 16, Li et al. [104] proposed a novel transparent reflection-type metasurface to achieve high transmission of visible light and NFF of microwave, demonstrating its potential for WEH applications. Its NNF transmission efficiency can reach more than 60% of the metasurface based on good conductor materials. The relative bandwidth with 50% transmission efficiency is 34.5% at 4.9–6.9 GHz. This work provides the promising potential to efficient WEH in glass curtain walls, indoor glass partitions, and other scenarios. For multiple functionalities, metasurfaces composed of building blocks that can be controlled are necessary. A holographic meta mirror that implements proper phase-profile distributions essential to maintain the focal spot dynamically is designed in ref. [33]. The reconfigurability mechanism introduced in the meta mirror through the incorporation of voltage-controlled electronic elements validates the concept of a reconfigurable focusing mirror where the single or multiple focal points can be set at different positions by electronically varying the phase profile of the metasurface given.

**Figure 17:**
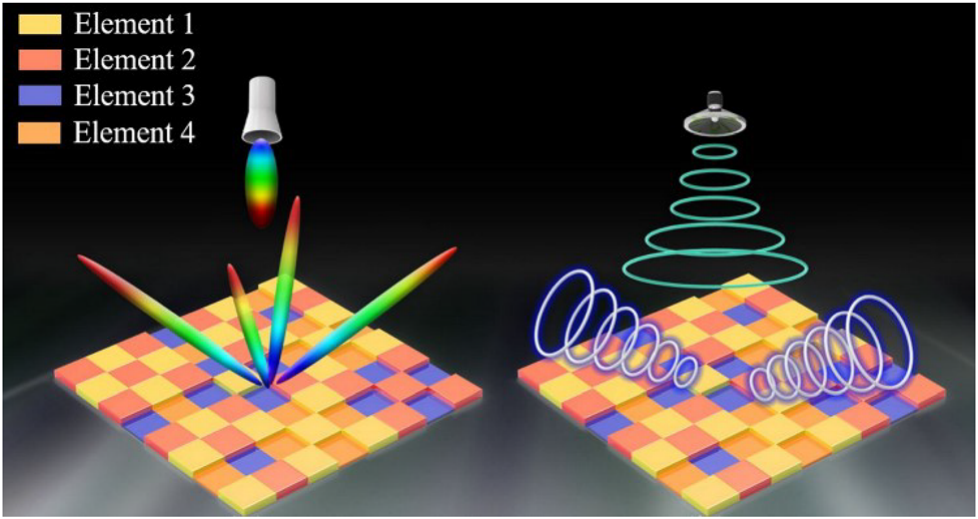
Schematic of the patterned MM and the corresponding EM scattering patterns at 6.0 GHz and acoustic ones at 6860 Hz under normal incidence [105]. Reprinted from [105], with the permission of American Chemical Society Publishing. © 2019, American Chemical Society.

The development of multi-functional metasurfaces provides more technical means for SWIPT. Zhang et al. [105] proposed a bifunctional digital coding metamaterial to engineer the propagation behaviors of EM and acoustic waves simultaneously and independently in Figure 17. Four kinds of rigid pillars with various material properties are employed to serve as 1 bit reflection-type digital meta-atoms with antiphase responses in both frequency spectra, thus offering the opportunities for independent field control as desired. The MM demonstrates excellent performance of scattering manipulations from 5.7 to 8.0 kHz in the acoustic region and 5.80–6.15 GHz in the microwave region. This work provides a solution for EM communication and acoustic energy supply for SWIPT. The research on multi-functional metamaterials for hybrid energy harvesting will make SWIPT more practical.

In this section, we mainly review the metasurfaces in WPT and WEH. EM waveS have the dual physical characteristics of information and energy. Therefore, the manipulation of EM waves is also the manipulation of information and energy. Metamaterials have attracted great interest in the emerging field of wireless charging. Focusing beam metamaterials can greatly improve the electromagnetic energy in the characteristic area. Non-diffractive beam metamaterials are expected to improve the distance of energy transmission. Relay-enhanced metamaterials can be used as an intermediate device to enhance the utilization of energy. These metasurfaces in WPT can improve the efficiency of the energy transmission of the WPT system. On the other hand, the metasurfaces in WEH can improve the efficiency of the wireless energy harvesting. Rectifying metamaterials combines metamaterials with rectifier circuits to achieve direct conversion of energy. For hybrid harvesting metamaterials, the collection of energy is not limited to single electromagnetic energy, but a variety of different mixed energy, which can improve the stability and durability of energy supply for wireless devices. The advantages of metamaterials for WPT and WEH are summarized in Table 2.

**Table 2:** The advantages of metamaterials for WPT and WEH.

Type of metamaterials	Advantage
Focusing beam metamaterials	Increase the intensity of the wireless energy
Non-diffractive beam metamaterials	Increase the distance of energy transmission
Relay-enhanced metamaterials	Enhance the utilization of the wireless energy
Rectifying metamaterials	Convert EM energy to DC
Hybrid harvesting metamaterials	Harvest different types of energy

## 4 Metamaterials for SWIPT

SWIPT is an effective strategy for integrating sustainable energy acquisition in the Internet of everything communication network and supporting new directions for information sharing and green energy. Many works focus on metamaterials for WIT, WPT, and WEH. However, very few are used for SWIPT. This section will summarize the potential metamaterials used in SWIPT and their future. The rapid advancement of wireless communication systems has prompted improved channel capacity and multi-mode communication. In the SWIPT system, the metamaterial is needed to make a trade-off between energy and information transmission to achieve a more efficient transmission rate. Separate reception of wireless power and information in frequency, polarization, beam, space-time has recently been proposed. In this section, we will explain the metamaterials for SWIPT in detail.

The major problem of SWIPT antenna is how to obtain energy and information simultaneously and minimize the interference between them. Current reports on SWIPT antenna design focus on circular polarization, multi-port, multi-band, rectified antennas and resource optimization algorithms for energy and information reception [106, 107]. Wagih et al. [108] proposed an SWIPT antenna consisting of a dual-port patch antenna and a rectifier circuit. This work uses a lower frequency band to transmit downlink energy to the wireless device and a higher frequency to send information in the uplink to the base station for simultaneous communication and energy transmission. Alternatively, the proposed antenna is fabricated by flexible materials and can be used in the human implantable. A rectifier antenna based on a highly isolated hybrid coupler for SWIPT has been proposed by Lu et al. [109]. The proposed antenna consists of a dual-port loop antenna, coupler, matching network and rectifier with multi-polarization and high efficiency.

### 4.1 Frequency manipulation metamaterials

**Figure 18:**
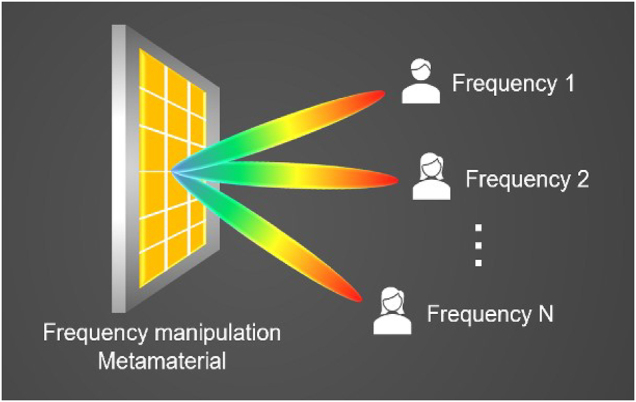
Frequency manipulation metamaterial.

**Figure 19:**
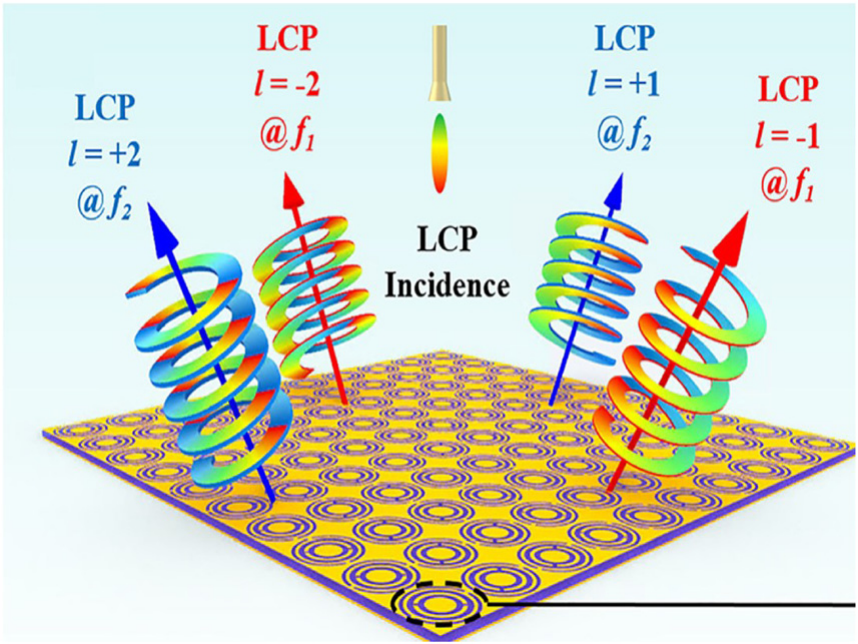
Independent phase controls at two frequencies [110]. Reprinted from [110], with the permission of AIP Publishing.

The frequency manipulation metamaterial plays a significant role in realizing systems that require the same aperture work at different frequencies, as shown in Figure 18, which are unachievable with conventional reflect arrays. Frequency is one of the fundamental properties of EM waves. The size of the metamaterial unit determines it. Recently, some works focused on the frequency manipulation metamaterials, which can flexibly switch working states at multiple frequencies to different needs. In ref. [110], one metamaterial unit, a double C-shaped slot resonator and a modified double C-shaped resonator in Figure 19, is proposed to achieve 2π reflection phase modulations at 9 and 13 GHz, respectively. The metamaterial unit provides an effective way to manipulate the frequency of EM waves. Similarly, Liu et al. proposed a dual-frequency metamaterial to generate dual-beam in different radiation directions at two distinct frequencies [111]. The metamaterial consists of two perfect half plates, an I-shaped bar and a split ring resonator, which can control the reflection phases at two distinct frequencies.

**Figure 20:**
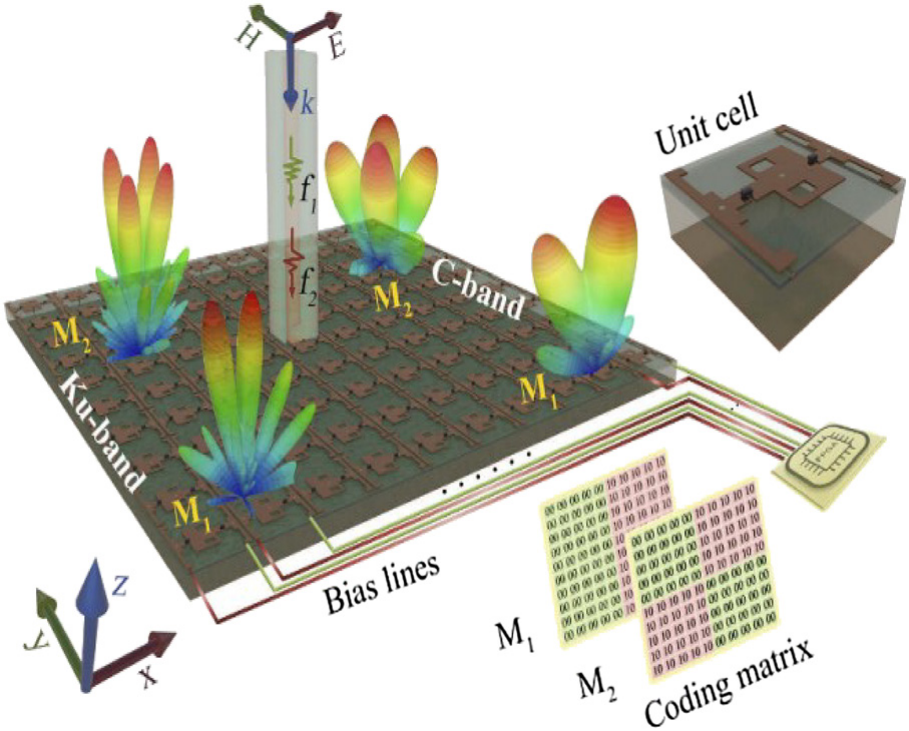
Conceptual illustration of the proposed dual-frequency multi-bit programmable metasurface from [14]. Reprinted from [14], with the permission of optical Society of America Publishing.

Active metamaterial paves the way to manipulate EM waves in real-time by incorporating tunable materials or components with versatile EM functionalities [55]. Zhang et al. designed a 1 bit programmable metasurface with an FPGA to perform completely independent real-time reconfigurability functions in C and X bands. The proposed metasurface can essentially enhance the information capacity, bringing new degrees of freedom in achieving versatile tunable functionalities. In ref. [14], a multi-bit dual-frequency programmable metasurface is proposed for real-time control of EM waves in the C-/Ku-band, as shown in Figure 20. The dual-frequency metasurface is controlled by the biasing voltages of the varactor diodes and PIN switches; hence, the diverse EM functions can be obtained as needed. Active tunable dual-frequency metasurface has tremendous advantages over the single band and passive metasurface. They increase the system capacity to transmit energy in two widely separated frequencies and enable real-time control of EM waves, beneficial for numerous applications in the modern wireless communication system.

**Figure 21:**
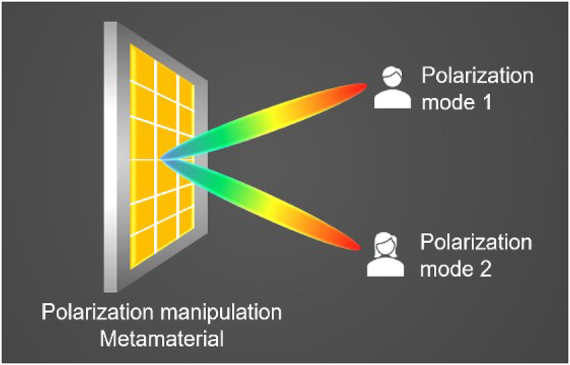
Polarization manipulation metamaterial.

These works provide a new solution for the realization of SWIPT. Some routers can work at different frequencies, which provides the feasibility for SWIPT: one frequency state is used to communicate, and the other is used to supply energy, which is called the frequency manipulation SWIPT. Different operating frequencies can be regarded as independent energy channels. The frequency manipulation SWIPT technology utilizes the diversity of spectrum to solve the problem of charging wireless communication devices.

### 4.2 Polarization manipulation metamaterials

Various approaches have been proposed to develop dual-polarization metamaterials that enable independent control of EM waves with different polarizations [112]. Typically, EM waves can propagate in two orthogonal polarization modes. EM waves in polarization mode 1 are independent of orthogonal mode 2. EM waves can only be received by receiving antennas with the same polarization mode. In this way, as shown in Figure 21, the energy carried by EM waves can charge wireless communication devices with different polarization modes. Different polarization modes can be flexibly switched to meet energy and information transmission.

**Figure 22:**
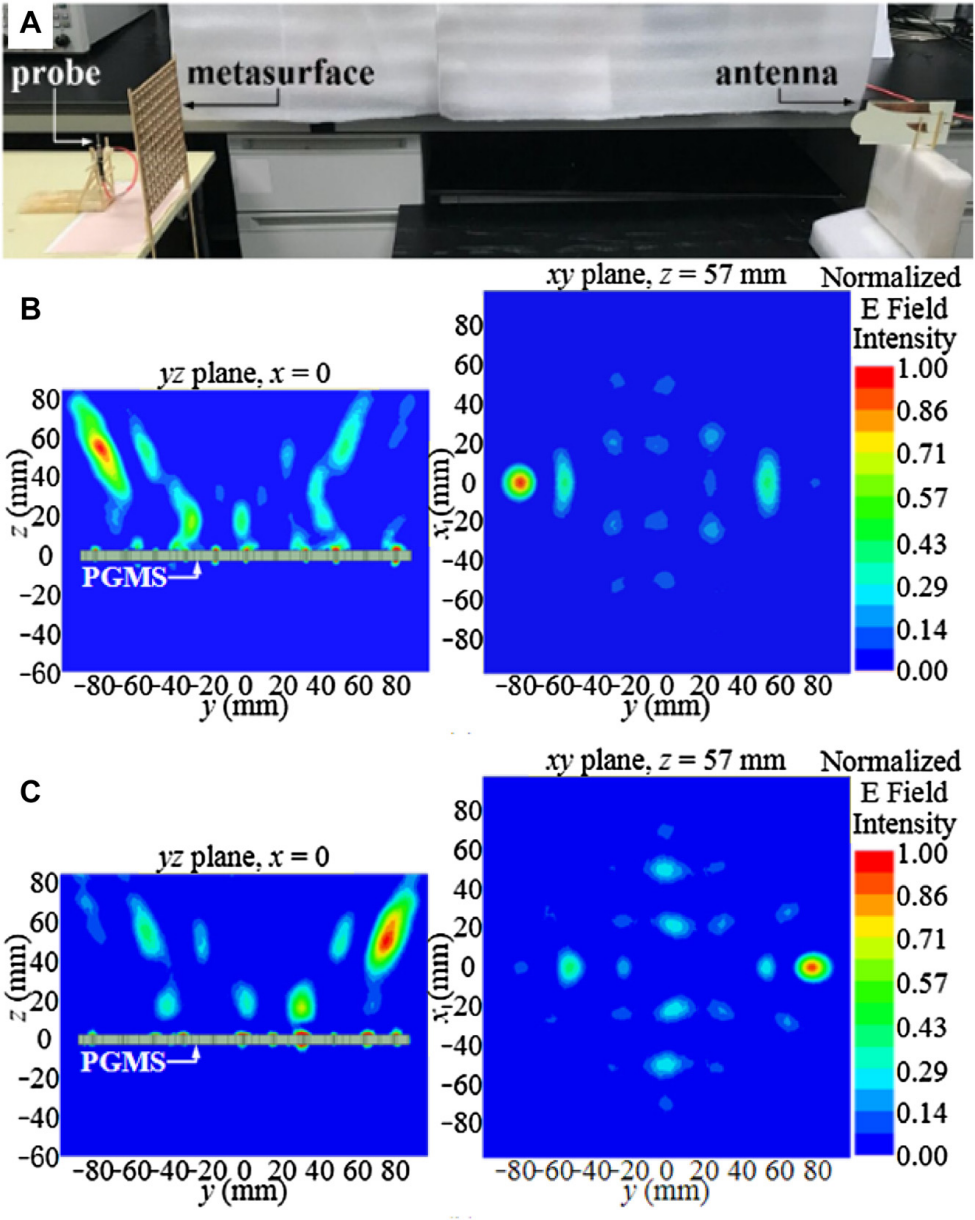
Polarization-controllable metasuface. (A) Polarization manipulation metamaterial. (B) and (C) The spatial distributions of the simulated normalized scattering electric field intensity at 14 GHz, for the *x*-polarization and *y*-polarization [113]. Reprinted from [113], with the permission of IEEE Publishing.

Wu et al. proposed a focusing MS with a polarization-controllable focus in the Ku-band [113]. The MS in Figure 22 is based on a single-layer dielectric structure consisting of cross-shaped dielectric elements. Without the use of any metallic unit, the dielectric element shows a high transmission magnitude of up to 0.95 and a nearly full phase-control coverage of about 330°. Moreover, the element is polarization-sensitive, capable of providing different phase gradients for the orthogonally polarized incident waves. Two spatially separated focuses can be obtained at the two orthogonal polarizations modes, and the focus positions can be independently controlled by tuning the lengths of the two orthogonal arms of the cross-shaped dielectric elements. Because of its independently controllable multi-polarization mode and multi-focus characteristics, the proposed MS can be applied to transmit information and energy to wireless communication devices in WPT systems.

Metamaterial, which works in two different polarization modes, is also a candidate for SWIPT: different polarization modes can be applied to transmit information and energy, called polarization manipulation SWIPT. Two orthogonal polarization modes do not affect each other. The polarization manipulation SWIPT technology utilizes the orthogonality of polarization modes, in which information and energy can be transmitted to wireless devices simultaneously. Programmable metasurfaces can achieve dynamic and real-time control of EM waves. The phase profiles of orthogonally polarization EM waves are mutually coupled in current works, or programmability can only be achieved under a specific polarization. Reference [112] proposed an intelligent sensing metasurface to achieve self-defined digital coding patterns for the *x*- and *y*-polarizations. The dual-polarization programmable metasurface can provide dual effective transmission channels for information and energy. The equilibrium ratio of information and energy can be dynamically controlled.

**Figure 23:**
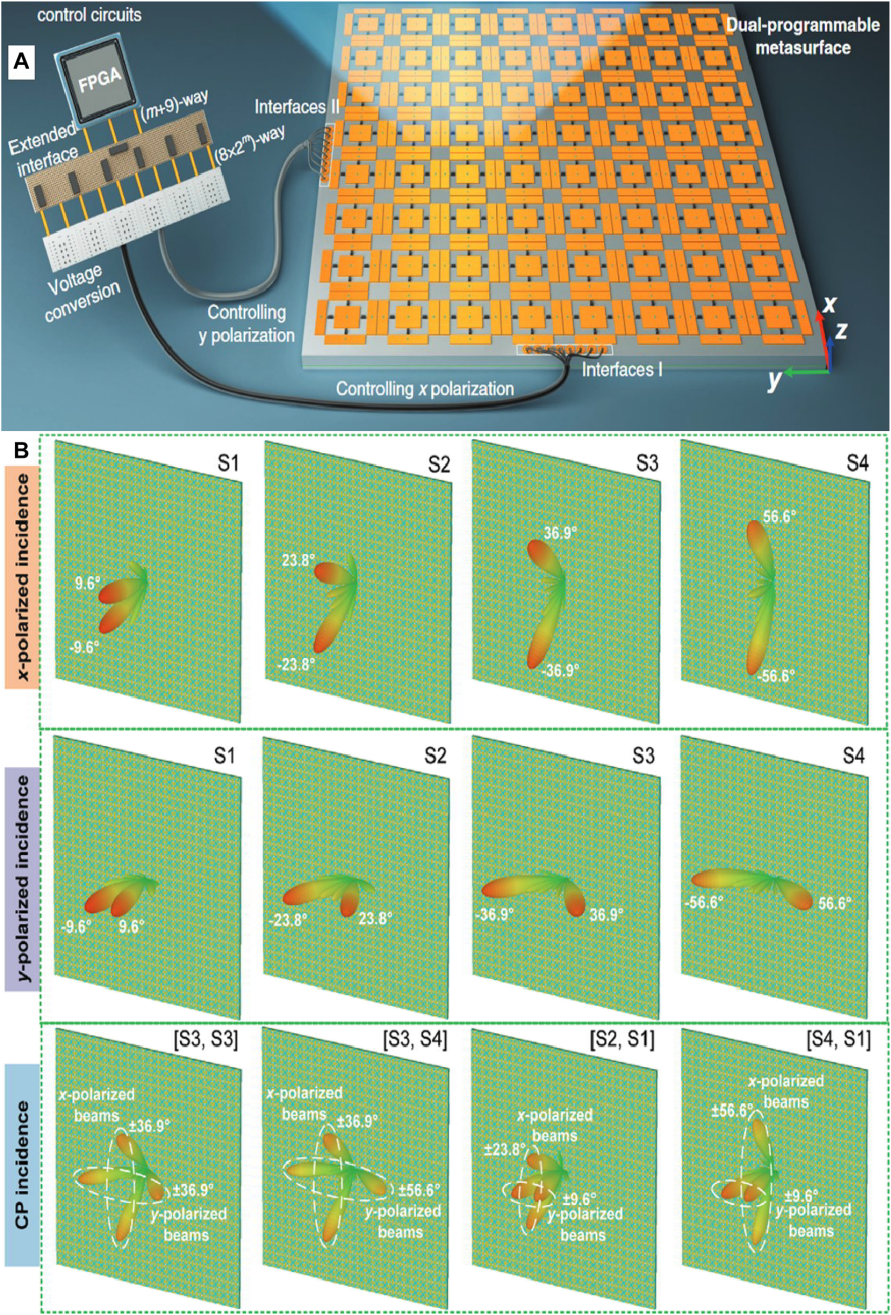
FPGA programmable metasurface. (A) PDPM for real-time and independent control of dual-polarization EM waves. (B) Different patterns at multi-polarization [114]. Reprinted from [114], with the permission of WILEY Publishing. © 2020 WILEY‐VCH Verlag GmbH & Co. KGaA, Weinheim.

**Figure 24:**
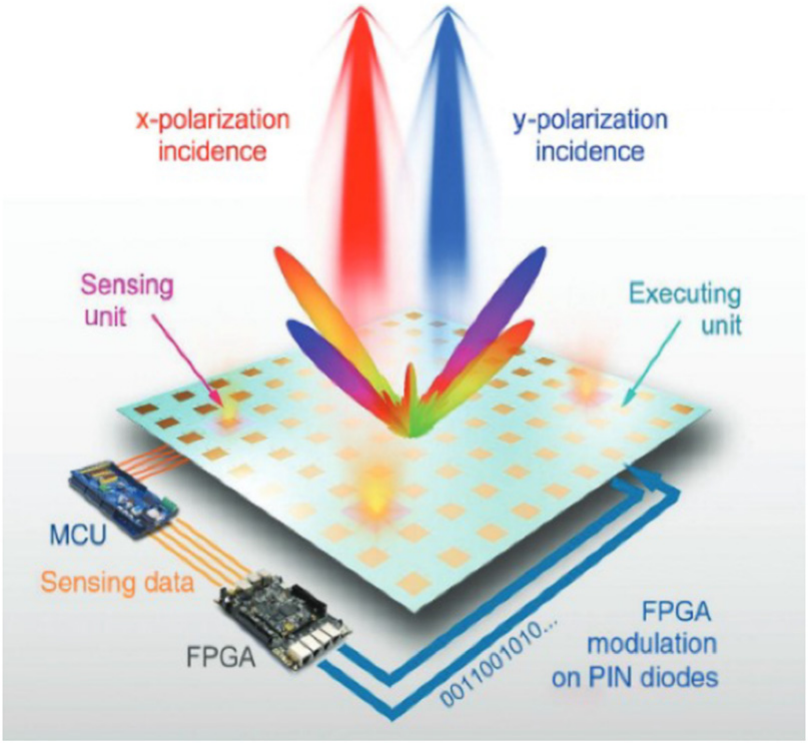
The schematic of the dual-polarization smart sensing metasurface [115]. Reprinted from [115], with the permission of WILEY Publishing. © 2020 WILEY‐VCH Verlag GmbH & Co. KGaA, Weinheim.

Therefore, achieving independent and real-time controls of orthogonally-polarized EM waves via FPGA is attractive for many applications. In Figure 23, Zhang et al. [114] proposed a dual-polarization 2 bit programmable metasurface. The dual-polarization metasurface was implemented by independently integrating varactors, programmed with two independent coding sequences to control the *x*-polarization and *y*-polarization waves. Bao et al. [115] presented a digital coding dual-polarization metasurface to manipulate the reflected phase in the *x*-polarization and transmitted phase in the *y*-polarization independently with the different bias voltages of diodes controlled by FPGA in Figure 24. The dual-polarization metasurface further increases the capability of information and energy in wireless communication systems. It opens up avenues for realizing more advanced and integrated multi-functional devices and systems with two independent polarization channels, which can be applied in future SWIPT systems.

### 4.3 Holographic beam metamaterials

**Figure 25:**
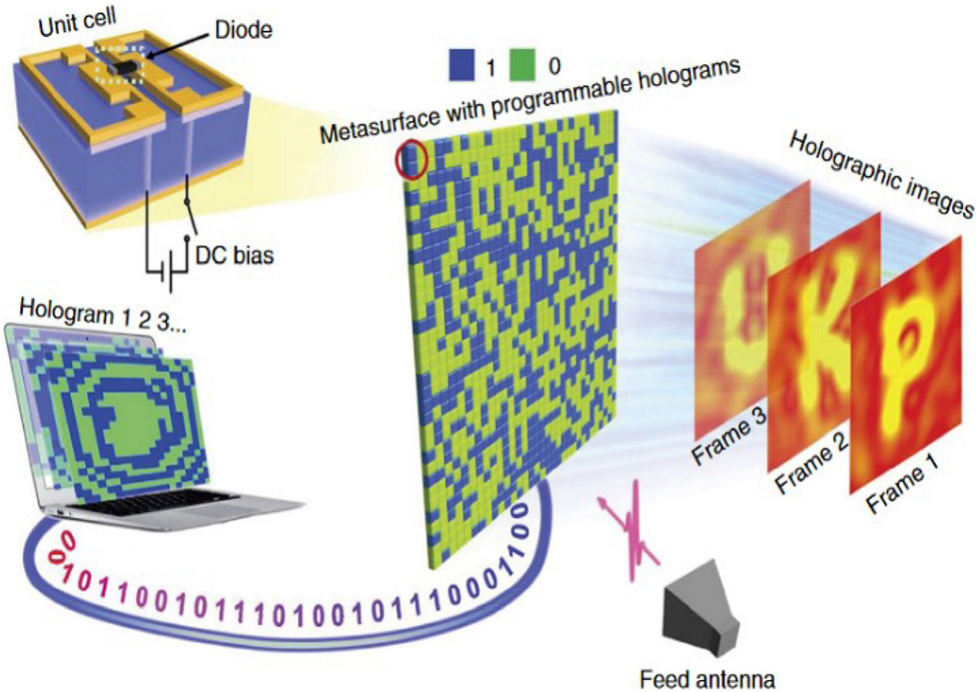
The dynamic holographic metasurface [117]. Reprinted from [117], with the permission of Nature Communications Publishing. © 2017, The Author(s).

The holographic beam can flexibly change the EM energy distribution in space and contribute to the three-dimensional energy supply. Holographic metamaterials can construct even more exotic EM beams. This technology enables communication in areas with weak energy and areas with strong energy as energy harvesting. Reference [116] presented an efficient holographic metasurface. It can form a diffraction-limited focal spot at a distance of 10 cm. The proposed NFF metasurface has high antenna efficiency and can find application as a compact source for Fresnel-zone WPT and remote sensing schemes. Li et al. [117] introduced the concept of a reprogrammable hologram based on 1 bit coding metasurfaces in Figure 25. The state of the coding metasurface units can be switched between “0” and “1” by electrically controlling the loaded diodes. The reprogrammable hologram metasurface can be readily extended to exhibit multiple bits and both phase and amplitude modulations. Different desired holographic images can be realized in real-time by a coding metasurface. The proposed reprogrammable hologram metasurface is a key for future intelligent information and energy system.

In ref. [118], a novel WPT scheme was proposed in the radiative near-field region, based on machine vision and dynamically reconfigurable holographic metasurface aperture capable of focusing energy to multiple spots simultaneously. The reconfigurability enables to achieve varying focusing characteristics for the same aperture. Firstly, the transmitter estimates the three-dimensional spatial coordinate of multiply users based on binocular vision. Secondly, the desired field distribution of the metasurface aperture to create focuses at the position of multiple users is calculated by analyzing three-dimensional spatial coordinates, which equivalents to treat the focused points as some fictitious point sources. Thirdly, by using the coaxial feed into the metasurface aperture, the magnetic field of the reference model can be realized. Lastly, the layout of the metamaterial elements and their tuning states is determined using holographic design principles to assess the required phase distribution of the aperture. This concept above can provide real-time and intelligent WPT for multiple users simultaneously with higher transmission efficiency.

### 4.4 OAM vortex beam metamaterials

**Figure 26:**
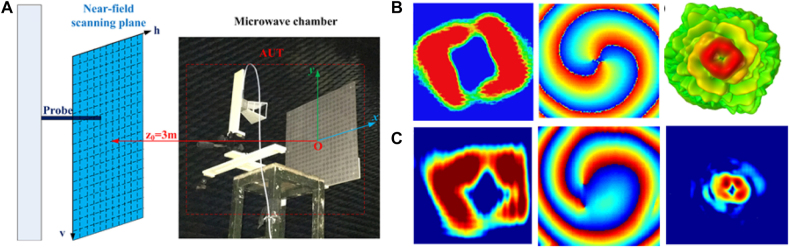
OAM vortex beam metasurface. (A) The reflective metasurface for orbital angular momentum vortex wave. (B) and (C) Simulation and measured results of the amplitudes, phases and patterns, respectively [120]. Reprinted from [120], with the permission of AIP Publishing.

OAM vortex beam can enhance the diversity of the beams to increase energy transmission channels. This part will explain the EM beam manipulation metamaterials in detail. The channel capacity of wireless communication systems is reaching the theoretical limit. Therefore, expanding new channel forms has become the focus to improve the channel capacity of wireless communication systems. OAM vortex beams have natural orthogonal characteristics between different OAM modes, providing extra information capacitance [119]. In other words, any OAM vortex beam is independent of others and does not affect each other. The information and energy of one OAM mode cannot be received by other modes, which simultaneously improves the channel capacity and security. In ref. [120], a reflective metasurface was designed to generate an OAM vortex wave in Figure 26. The proposed metasurface can generate OAM vortex waves with different modes simultaneously. The proposed method paves the way to generate the OAM vortex waves for WPT applications. For the transmitter, the energy can be transmitted by different OAM vortex beams to wireless devices with the same OAM mode, which improves equipment recognition, especially in a complex multi-device WPT system. Lee et al. [121] introduced a cyclic group symmetric metasurface composed of tapered arc nano-rods and explored how azimuthal angular distribution of total phase determines the feature of spin-dependent beam separation. Relation of cyclic group symmetry property of metasurface and the generated vortex beam profile is examined in detail by experimental measurement and analysis in terms of partial-wave expansion and non-constant azimuthal gradient of the total phase. The capability of spatial beam profiling by spin-dependent beam separation in vortex beam generation has an important implication for spatial demultiplexing in optical communication utilizing optical angular momentum mode division multiplexing and optical SWIPT.

### 4.5 Space-time coding metasurfaces

**Figure 27:**
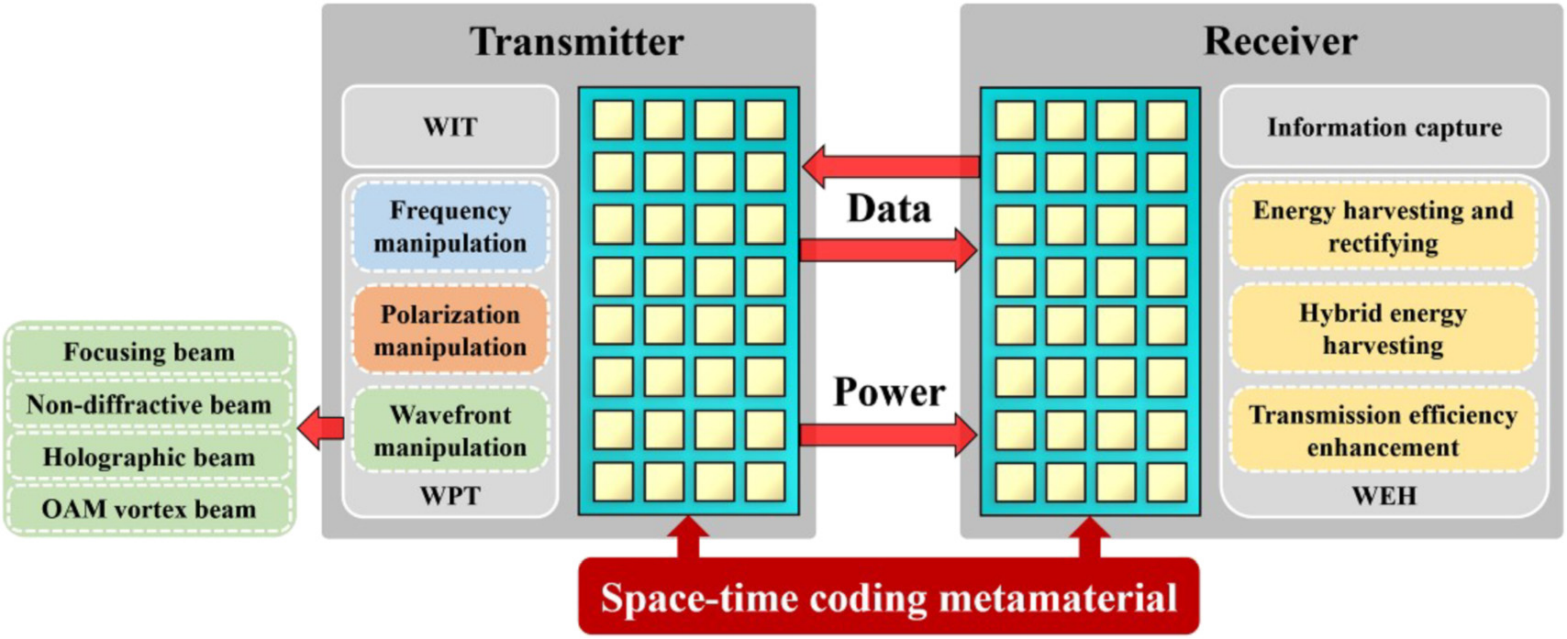
The STCM for SWIPT.

Space-time coding metasurface (STCM) can manipulate the propagation direction and harmonic power distribution of electromagnetic waves, making them suitable for space- and frequency-division multiplexing [71]. STCM is a potential technology to encode and transmit information for wireless communication systems [39, 122, 123]. By introducing the time dimension, STCM improves EM waves’ manipulation to realize space-time division multiplexing. By encoding space–time-coding matrices through multiple channels, information can be directly transmitted to multi-user at different locations simultaneously. Recently, the concept of STCM has been presented. The codings “0” and “1” can characterize the element’s different amplitudes, phases, and polarization characteristics. STCM demonstrates strong vitality in wireless communications, as shown in Figure 27. It can achieve programmable EM waves in real-time by integrating with FPGA. On the other hand, the digital coding strategy bridges the physical and digital worlds, making the metamaterial realize direct information processing. With these outstanding advantages, STCM has shown great potential in wireless communications and SWIPT systems [105, 124, 125].

STCM allows it to control EM waves in real-time and implement many different functionalities in a programmable way. However, current configurations are only space-encoded and do not exploit the temporal dimension [124, 126, 127]. Recent advances in metamaterials and metasurfaces have provided an unprecedented degree of freedom to manipulate EM waves at sub-wavelength scales, giving rise to vastly enhanced nonlinearities, thus offering new possibilities to control the intensity, phase and polarization states of the induced harmonics [128]. STCM has been proposed to simultaneously achieve efficient frequency conversion and harmonic control; they show considerable potential for many EM applications such as wireless communications. However, achieving flexible and continuous harmonic wavefront control remains an urgent problem.

**Figure 28:**
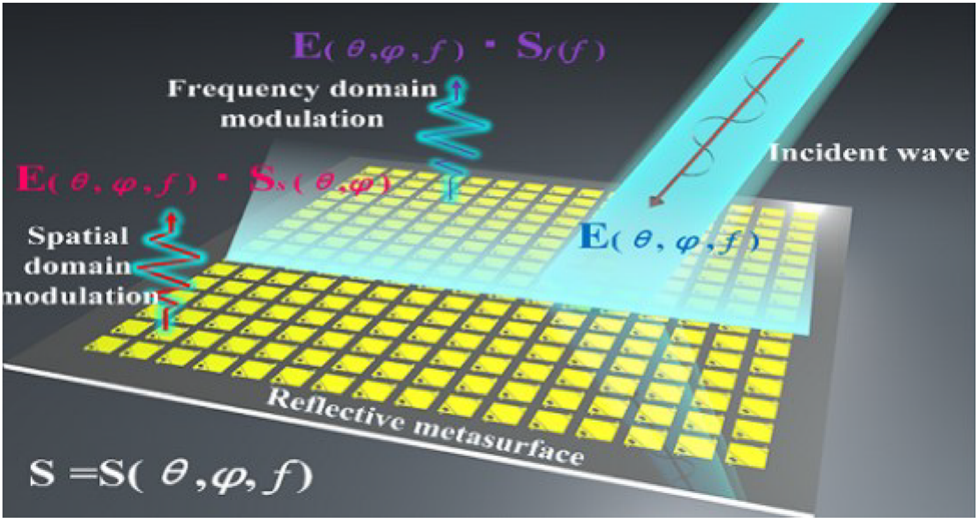
Frequency- and spatial-domain reconfigurable metasurface [129]. Reprinted from [129], with the permission of American Chemical Society Publishing. © 2020, American Chemical Society.

In ref. [129], Liu et al. proposed a general frequency- and spatial-domain reconfigurable metasurface (FSRM), shown in Figure 28, to manipulate EM waves and realize reconfigurable functions in multi-frequency bands. In the frequency domain, FSRM can convert different linearly polarized (LP) incident waves into left- and right-hand circularly polarized reflected waves, in which PIN diodes are used to switch the polarization conversions in different frequency bands. When the polarization direction of the incident LP wave is 45° from the +*x*-axis, the FSRM manipulates the incident waves as a 1 bit programmable metasurface in the spatial domain. Two-dimensional beam scanning, vortex beams with orbital angular momentums, and specific beams with desired transmission directions are demonstrated via real-time adjustment of the digital coding state. The design strategies presented in this work can also tailor EM waves in many other intriguing ways for further exploration.

**Figure 29:**
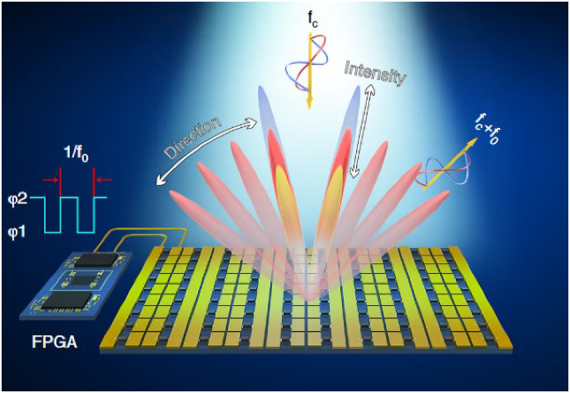
Schematic diagram of the time-domain digital coding metasurface [130]. Reprinted from [130], with the permission of Springer Nature Publishing. © 2018, The Author(s).

Dai et al. [130] designed and experimentally characterized a reflective time-domain digital coding metasurface, with independent control of the harmonic amplitude and phase in Figure 29. As the reflection coefficient is dynamically manipulated proactively, a significant conversion rate from the carrier signal to the harmonic signal is observed. What’s more, by encoding the reflection phases of the meta-atoms, beam scanning for multiple harmonics can be implemented via different digital coding sequences, which paves the way for efficient energy and information transmission for applications in SWIPT areas.

**Figure 30:**
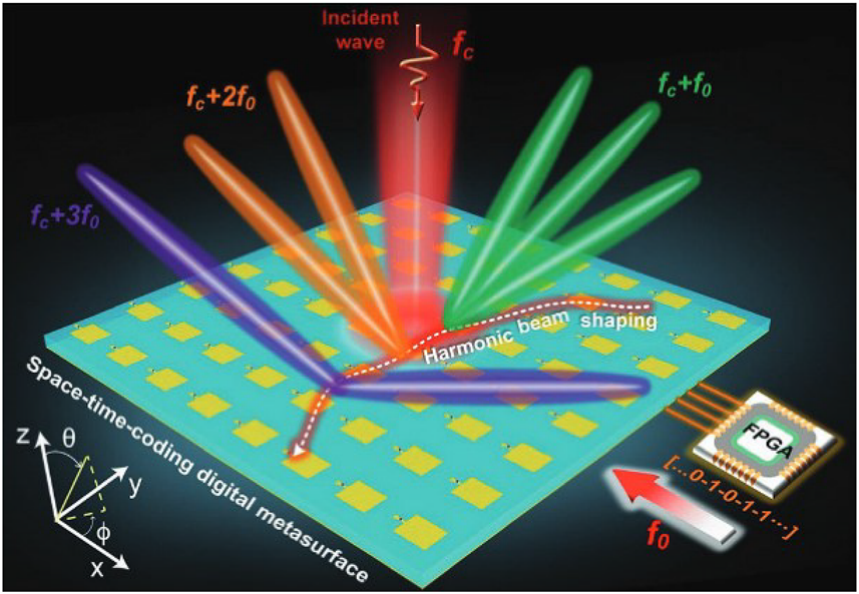
Conceptual illustration of the joint multi-frequency beam forming and steering via an STCM [131]. Reprinted from [131], with the permission of John Wiley and Sons Publishing. © 2020 Wiley‐VCH GmbH.

STCMs have been successfully applied to advanced beam-manipulations in both the spatial and spectral domains. In ref. [131], the idea was proposed of joint multi-frequency beam forming and steering of scattering patterns via STCMs in Figure 30. Several illustrative numerical examples, including multi-beam, diffuse, and OAM-type scattering patterns, were presented and discussed. A 2 bit prototype demonstrated good agreement between theoretical predictions and measurements. This enables a variety of intelligent functionalities, including self-adaptive and cognitive EM waves manipulation, which may find exciting applications within the SWIPT.

STCM has recently attracted significant attention for the additional degree of freedom they offer in manipulating EM waves in both space and time domains. STCM can create independent manipulations of arbitrary harmonics and wave behaviors, bringing considerable potential to the SWIPT system. Zhang et al. present Fourier operations on a time-domain digital coding metasurface and propose a principle of non-linear scattering-pattern shift using a convolution theorem that facilitates the steering of scattering patterns of harmonics to arbitrarily predesigned directions. Introducing a time-delay gradient into a time-domain digital coding metasurface allows us to deviate anomalous single-beam scattering in any direction successfully, and thus, the corresponding formula for the calculation of the scattering angle can be derived. This work paves the way for controlling energy radiations of harmonics by combining a non-linear convolution theorem with a time-domain digital coding metasurface, thereby achieving more efficient control of EM waves.

In this section, we summarize the usefulness of metamaterials for SWIPT. Similar to the information channel in wireless communication systems, information and energy will also be transmitted in different transmission channels in the future communication systems. Therefore, exploring new energy transmission multiplexing technology has become the key research. The frequency, polarization and shape of EM wave can used to realize the multiplex transmission of wireless energy. The advantages of metamaterials for SWIPT are summarized in Table 3.

**Table 3:** The advantages of metamaterials for SWIPT.

Type of metamaterials	Advantage
Frequency manipulation metamaterials	Use different frequencies as different channels
Polarization manipulation metamaterials	Transmit wireless information and energy in different polarization modes
Holographic beam metamaterials	Utilize the EM energy distribution to realize SWIPT
OAM vortex beam metamaterials	Natural orthogonality of different OAM modes
Space-time coding metasurfaces	Achieve SWIPT in space-domain and time-domain in the digital coding form

## 5 Challenges and future

In SWIPT systems, the performance of the transmitting and receiving antennas is one of the critical parameters [53]. The goal of a transmitting antenna for SWIPT systems is to generate a beam of information and energy flow simultaneously, where the beam for transmitting information should have characteristics including high gain and high channel capacity. On the other hand, the beam to transmit energy should be focused, non-diffractive and have high conversion efficiency. The SWIPT transmitting antenna should have an independently controllable beam for energy and information. Next, the receiving antenna has also attracted strong interest for SWIPT, which can receive as much energy as possible to power the device and extensive information for the back-end device to process simultaneously. It should have circular polarization, high reception efficiency, wide-angle range and high EM to DC conversion efficiency. In addition, a complete SWIPT system requires an energy storage module and an information processing unit to simultaneously process the energy and information acquired by the receiving antenna [132].

Transmission distance is one of the significant concerns in SWIPT, but EM energy transmission is limited in terms of its coverage range due to the inverse-square law. Limited by the performance of materials and devices, there is a bottleneck in the efficiency of the SWIPT system. There is unavoidable energy loss in the process of energy transmission and reception. The energy of rectifying and management is a significant challenge for the receiving end. Research and development of novel materials and devices will enhance the current technologies. GaN is a material with excellent application prospects, and it is expected to be used to improve the performance indicators of active devices. With the novel GaN devices, the efficiency of wireless power transmission and harvesting will be significantly improved.

The future wireless communication system is expected to meet unprecedented performance requirements to support our highly digital and global information-driven society [39, 68]. For the SWIPT system, there are many challenging problems. In the SWIPT system, reasonable modulation of the operational status of each device is a critical issue. Dynamic modulation of energy supply will reduce energy loss and improve system efficiency, which is more environmentally friendly. Therefore, artificial intelligence (AI) technology can be introduced to realize the intelligent, autonomous, and dynamic control of the SWIPT system. Reconfigurable AI-enabled STCM is the product of combining artificial intelligence and metamaterials. STCM has attracted extensive attention among many potential technologies, leading to a proliferation of studies in wireless communication. The STCM-based wireless communication system and AI-enabled technologies, two of the promising technologies for the sixth-generation networks, interact and promote with each other, striving to collaboratively create a controllable, intelligent, reconfigurable, and programmable wireless propagation environment. AI-enabled technologies would be integrated to maximize the usefulness of STCM. The reconfigurable STCM simultaneously uses the dual-channel of time and space, bringing more possibilities for developing the SWIPT system. Utilizing the extraordinary characteristics of STCM, the overall performance will also be improved.

## 6 Conclusions

This review summarized the most recent metamaterial works in SWIPT technology. Focusing on SWIPT systems, our work provides guidance on various metamaterial-based models and system designs for WIT, WPT and WEH. Metamaterials significantly improve WIT systems’ information transmission, reception, multiplexing and processing capabilities, making wireless communication systems more intelligent. Meanwhile, the practicality of metamaterial-based WPT technology has been dramatically improved. This paper highlights some metamaterial-based WIT and WPT technologies that can also be applied to SWIPT systems, such as manipulating frequency, polarization, and beam, which requires researchers to have a richer image of the application of these technologies. The rapid development of metamaterials will play an essential role in SWIPT: the generation and control of EM waves, the concentration and long-distance transmission of EM energy, the reception and utilization of EM energy, and the information transmission. In the era of 5G/6G communication, SWIPT technology is fundamental for energy and information transmission within numerous types of modern communications networks. As an epoch-making technology, SWIPT raises many novel research questions of interest and challenge, including resource allocation, communication and energy security, environmental protection, information sharing, reliability architectures and more. We hope that the technologies presented in this review will help stimulate future research in the exciting new area of energy and information cooperation in metamaterial SWIPT and pave the way for the future design and implementation of efficient and intelligent SWIPT systems.
